# Small Size of Recorded Neuronal Structures Confines the Accuracy in Direct Axonal Voltage Measurements

**DOI:** 10.1523/ENEURO.0059-21.2021

**Published:** 2021-08-02

**Authors:** Viktor János Oláh, Gergely Tarcsay, János Brunner

**Affiliations:** 1 Laboratory of Cellular Neuropharmacology, Institute of Experimental Medicine, H-1083, Budapest, Hungary; 2 János Szentágothai School of Neurosciences, Semmelweis University, H-1085, Budapest, Hungary

## Abstract

Patch-clamp instruments including amplifier circuits and pipettes affect the recorded voltage signals. We hypothesized that realistic and complete *in silico* representation of recording instruments together with detailed morphology and biophysics of small recorded structures will reveal signal distortions and provide a tool that predicts native, instrument-free electrical signals from distorted voltage recordings. Therefore, we built a model that was verified by small axonal recordings. The model accurately recreated actual action potential (AP) measurements with typical recording artefacts and predicted the native electrical behavior. The simulations verified that recording instruments substantially filter voltage recordings. Moreover, we revealed that instrumentation directly interferes with local signal generation depending on the size of the recorded structures, which complicates the interpretation of recordings from smaller structures, such as axons. However, our model offers a straightforward approach that predicts the native waveforms of fast voltage signals and the underlying conductances even from the smallest neuronal structures.

## Significance Statement

Patch-clamp instruments interfere with biological voltage signals, especially when the recorded structure is small. To overcome this limitation, we built a realistic model that considers both biological entities and recording instrumentation including amplifier and pipette, which introduce most of the distortions. We measured and simulated the electrical contributions of various instrumental components allowing not only the quantification of errors but also the reconstruction of native signals that would be generated in the excitable structure without instrumental distortion. We predicted the undisturbed behavior of a small axon from an inherently distorted recording. Altogether, we revealed that size of the recorded structures is critical for accurate voltage measurements, but these limitations can be overcome by including the accurate model of recording instruments.

## Introduction

Patch-clamp technique is affected by limitations that originate primarily from the physical properties of the recording pipettes (Hamill et al., 1981; Benndorf, 1995; Marty and Neher, 1995). Patch pipettes have significant resistance (R_pip_) and their glass wall represent a substantial capacitive surface (C_pip_) as well. Limitations can be reduced by optimizing the recording conditions (that is, with the reduction of the R_pip_ and C_pip_) and corrected by using compensatory mechanisms of the amplifiers. Under standard recording conditions in measurements from relatively large structures, such as neuronal somata, these optimizations and corrections can sufficiently reduce distortions to an acceptable level. Therefore, the difference between the recorded and native voltage signals are negligible during good current clamp (CC) conditions in most neuronal structures. However, the reduction of instrumental distortions could become inherently insufficient in cases where the recorded structures are small, such as most of the central synapses. Recording pipettes for small neuronal structures must have small tip, which inevitably results in larger R_pip_ values that substantial filters the recorded signals (Benndorf, 1995; Ying et al., 2002; Novak et al., 2013). Consequently, the fast voltage signals such as the action potentials (APs) are particularly vulnerable to signal distortion associated with direct recordings in small axonal structures. The shape of axonal APs is a key determinant of neuronal signaling that affects neurotransmitter release and short-term dynamics in synaptic connections (Katz and Miledi, 1967; Borst and Sakmann, 1999; Geiger and Jonas, 2000; Bean, 2007; Kawaguchi and Sakaba, 2015; Chao and Yang, 2019; Zbili and Debanne, 2019). This synaptic activity generates dynamic changes in the postsynaptic membrane potential that would be translated into AP firing of the postsynaptic neuron (Koch and Segev, 2000; London and Häusser, 2005; Silver, 2010). Therefore, accurate measurement of the membrane voltage is essential to understand fundamental mechanisms of the neuronal information flow. Recent developments of the recording apparatus allows collecting voltage signals from the finest axonal structures (Novak et al., 2013; Kawaguchi and Sakaba, 2015; Rowan et al., 2016; Vivekananda et al., 2017; Ritzau-Jost et al., 2021). However, the interpretation of these signals are still limited because signal distortions caused by the recording pipette and amplifier circuits remains elusive (Ritzau-Jost et al., 2021). We reasoned that as computational modeling allows the precise reconstruction of the native electrical behavior of the most complex neuronal structures (Alle et al., 2009; Branco and Häusser, 2011; Jayant et al., 2017; Kwon et al., 2017; Beaulieu-Laroche and Harnett, 2018), it should be similarly possible to simulate the behavior of the recording instruments.

With this in mind, we built and tested a realistic model that considers not only the biological structures but also amplifier and pipette features. Using this complex model, we simulated actual recording conditions with the aim of subsequent recovery of undistorted (native) signals by removing instrumental contributions from the model. Thus, this complex model allowed the correction of the distortions caused by patch-clamp recording instruments and the prediction of isolated biological signals. We tested the model by predicting the native AP waveform of a directly recorded small axonal varicosity. Our simulations showed that recording instrumentation not only filters the signal, but it directly interferes with native signal generation in small neuronal structures.

## Materials and Methods

Experimental procedures were made in accordance with the Institute of Experimental Medicine Animal Care Committee’s regulations (MÁB-7/2016, PE/EA/48-2/2020).

### Constraining the amplifier model

#### The model cell

Electrical components of the customized model cell (test #3 circuit, modified type 1U, Molecular Devices) were connected through conductive metal slots taken from a circuit breadboard allowing the change of circuit components without the need for soldering that would introduce variable stray capacitance (Extended Data Fig. 1-1). We used non-inductive, low-noise resistors: resistors either originally present in the 1U model cell, 10 and 500 MΩ, or Ohmite SLIM-MOX10203 series, 20–100 MΩ. Stray capacitance of each element of the test circuits (including the resistors and their slots) was characterized in VC mode by measuring the capacitive load associated with the introduction of the given circuit element. All capacitors were considered ideal, that is, without any resistive component.

#### Boosting unit

We implemented a simplified boosting unit (Sigworth, 1995) in which capacitors were fixed (100 and 120 pF) while resistors were directly fitted to reproduce the capacitive current response in the test #1 configuration. Late phase of the response profile (starting 24 μs after the stimulus onset) has particular importance because artefacts in that temporal domain can contaminate measured biological signals, therefore, this part of the signal was heavily weighted (850×) during the adjustment of the boosting unit.

#### VC capacitance compensation

Both CPf and CPs circuits were designed as described previously (Sigworth, 1995).

#### Stray capacitance of the CC circuit

**Figure 1.**
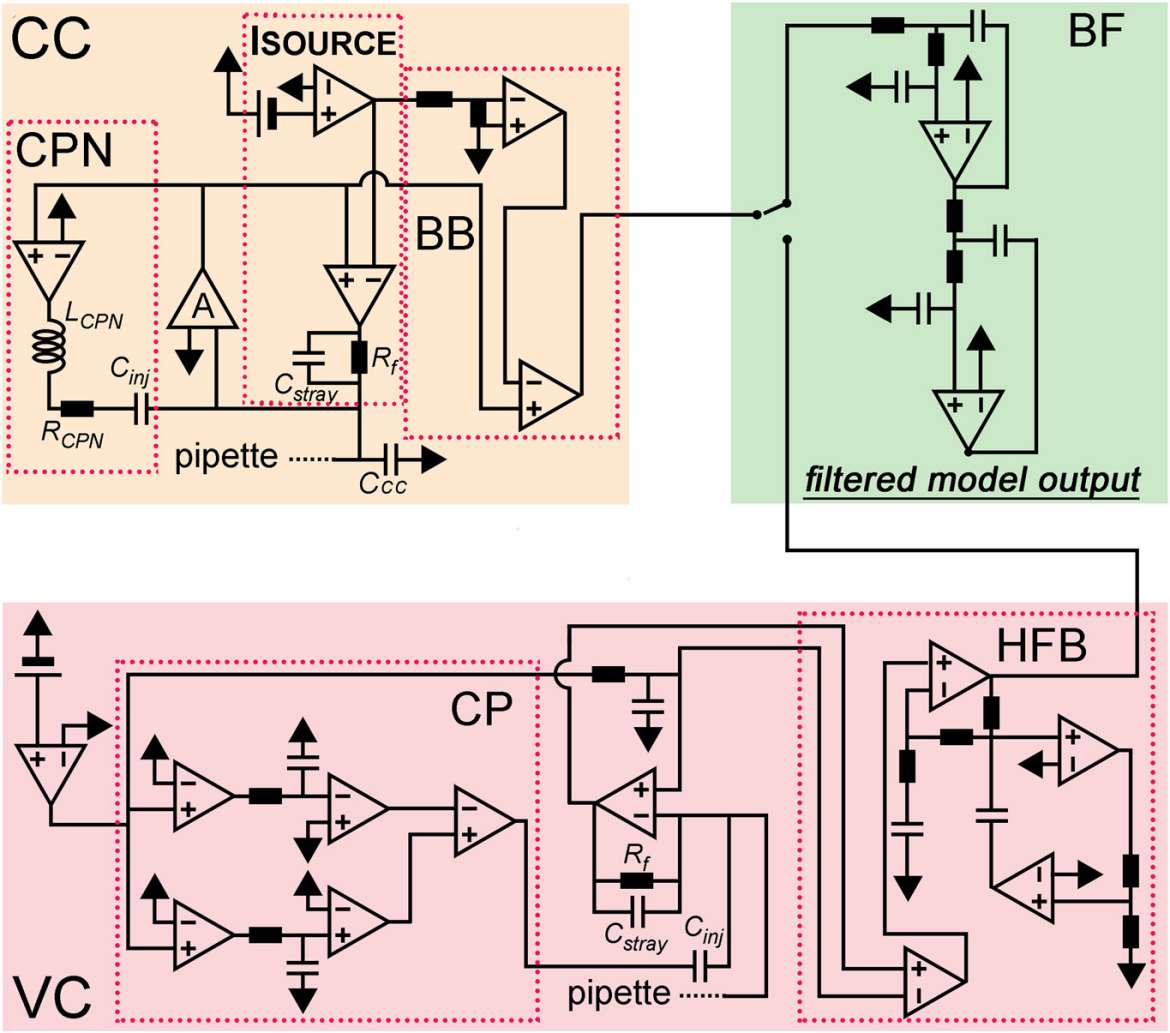
Model amplifier. Schematic circuit diagram of the model amplifier. The current clamp circuit (CC) consisted of a current source (ISOURCE), an idealized voltage buffer (A), capacitance neutralization (CPN), and bridge balance (BB) circuits. The voltage clamp circuit (VC) consisted of a resistive feedback amplifier with dual capacitance compensation (CP) and the high-frequency boost circuit (HFB). Outputs of the two amplifier modules are connected to a low-pass filter circuit (BF). The test configurations used to characterize the implemented amplifier components are shown in Extended Data Figure 1-1.

To optimize the circuitry of CC model, we performed measurements with test #3 circuit, where pipette parameters (10 MΩ, 2.8 pF) and the size of the cell-equivalent resistor (500 MΩ) were fixed, only cellular capacitance varied from 0.75 to 46.7 pF. We applied short current stimuli (−50 to −200 pA, 3 ms) to elicit voltage responses with the maximal attainable capacitance neutralization (CPN) or without CPN. Traces recorded in the absence of CPN allowed us to characterize the total capacitive load of the CC circuitry. The model most accurately reproduced the real voltage responses when a 0.76 pF stray capacitance was added at the amplifier input node (Fig. 1, C_CC_).

10.1523/ENEURO.0059-21.2021.f1-1 Extended Data Figure 1-1 Test circuits used for the characterization of the circuit components. Download Figure 1-1, TIF file.

#### CPN

**Figure 2.**
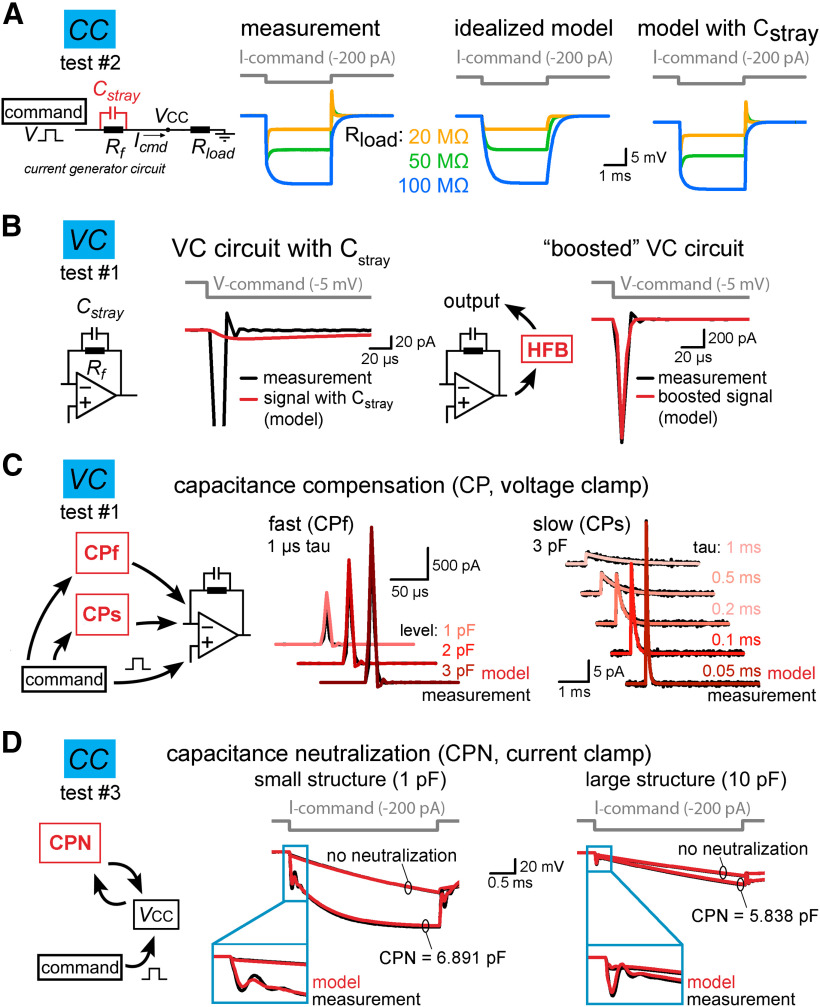
Implementation of individual amplifier components with realistic parameters. ***A***, The stray capacitance (C_stray_) associated with the current passing resistor (R_f_) was predicted by simulating the voltage responses recorded in test #2 configuration. C_stray_ acts as a capacitance neutralizing element in CC mode. Because the capacitance neutralizing effect of C_stray_ depends on the load resistance (R_load_) attached to the amplifier input (V_CC_), we tested three scenarios using 20-, 50-, and 100-MΩ resistors. The left traces show measured voltage responses evoked by brief current injection in the presence of different R_load_. Idealized (i.e., C_stray_ free) model responses are shown in the middle for comparison. Right, Model responses with 0.38 pF C_stray_ replicated the observed amplifier behavior. ***B***, C_stray_ of R_f_ slows down the current responses in the VC model (red trace, left panel). This effect is compensated in the actual amplifiers by using high-frequency boost circuit (HFB). The implementation of a HFB circuit in the model restored the response speed (red trace, right panel). ***C***, Capacitance compensation in the VC model (red) faithfully replicates the magnitude and the time course of amplifier-generated compensatory currents (black, test circuit #1) for both the fast (CPf) and slow (CPs) compensations. ***D***, Comparison of simulated (red) and recorded (black) voltage signals obtained with different capacitance neutralization (CPN) in CC mode from a small structure (middle panel) and from a large structure (left panel). Notice that the instrumental CC model faithfully replicates the neutralizing capability of the amplifier and the neutralization associated signal artefacts regardless of the applied CPN settings.

We implemented CPN in two steps. First, we added an idealized positive feedback loop, where the compensation through the C_inj_ can be modulated with the gain of an operational amplifier. This simple circuit representation was sufficient to reproduce the neutralizing capability of the real amplifier, that is, equal CPN settings resulted in the same level of compensation in the model as in the real experiments. Next, we reproduced the characteristic CPN-related stimulus artefacts that are present in typical CC measurements. We placed a resistor (R_CPN_) and an inductor (L_CPN_) to the CPN path (Fig. 1) and tuned their parameters by direct fitting of the model voltage responses to the experimental data (examples are shown in Fig. 2*D*).

#### Bridge balance (BB) compensation

To recreate the BB circuit, we created a reference signal from the command equivalent to the voltage drop caused by 1-MΩ load resistance (Fig. 1). Scaled version of that reference signal was then subtracted from the pipette voltage for the correction.

#### Bessel filters

We added filters to the amplifier model to match our actual biological recordings. We added an active linear filter consisting of two cascaded Sallen–Key filter stages (Fig. 1). Filter parameters were set according to an available filter design tool (https://tools.analog.com/en/filterwizard/) to produce output with four pole low-pass Bessel filter characteristics. All recordings were made with bypassed filtering mode, and we applied a 100-kHz lowpass filter throughout the simulations.

### Pipette parameter measurements

We used typical borosilicate glass capillaries (BF150-75-10, Sutter Instruments) to fabricate pipettes that are suitable for recordings from small axons. To implement these high-impedance pipettes into the model we measured their actual parameters. First, we assessed the actual pipette capacitance as a function of the tip distance by dipping known part of the recording pipettes into the recording solution. Pipette position was measured by the *x*-axis values of the motorized micromanipulator (SM5 controller with Mini unit, Luigs und Neumann). First, we recorded the total capacitance of the instrumentation in open circuit VC mode when the pipette was out of the solution. Then, we gently moved the pipette to a position where the tip intermittently reached the surface of the fluid characterized by the appearance of short conductive periods in the recorded VC signal. Then, the pipette was pushed forward to the solution to reach a position where the conductive state became stable (typically <5 μm forward movement). Starting from this tip position point, we systematically increased the length of the dipped part and quantified the capacitance in VC by integrating the first 50 μs of the transient response to a −20-mV voltage step. Integrated area was divided by the voltage step amplitude to convert the electric charge to capacitance.

To measure distribution of resistance along the pipettes, we broke off a known length of the pipette and measured the resistance of the remaining part in VC mode. First, we moved the pipette tip to a defined position under the objective and recorded its resistance. After withdrawal of the pipette, we broke the tip by gently touching it with a piece of lens cleaning tissue. The newly formed pipette tip was then positioned back to the reference position on the image. We determined the length of the broken pipette parts by reading the difference of the positioning motor. Resistance was measured in VC mode using −2- to −20-mV steps. We repeated the breaking process for each pipettes several times (1–8×, using 22 pipettes in total).

We visualized the outer/inner diameter ratio (R_OI_) of the recording pipettes using a grinding system that gradually cut them in half. This measurement allowed to avoid the optical distortions caused by the lens effect of the cylindrical glass. The recording pipettes (the first 8–12 mm from the tip) were embedded into epoxy resin on a microscope slide. Pipettes were longitudinally grinded with a coarse-grained aluminum-oxide abrasive disk (grit size = 600) until we reached the surface of the pipettes. Then, the grinding was occasionally interrupted to check the surface and cross-section of the pipette under a transmitted light microscope (Leica DM2500 microscope, 5–100× magnifications). As the plane of the pipette tip was approached, we switched to fine-grained abrasive discs (grit size = 6000). The images obtained from different cross-section levels were then used to measure the pipette diameters (Adobe Photoshop 5.0). The diameter data were included only from those focal planes for distinct segments of the pipette tips, where the outer diameter was the largest and the relative wall thickness was the smallest. We assumed infinite pipette wall resistivity in the model.

### Slice preparation and electrophysiology

Hippocampal slice was prepared from a 29-d-old Wistar rat. The animal was deeply anaesthetized with isoflurane. After decapitation, the 350-μm-thick slice was cut with Leica VT1200S vibratome in ice-cold cutting solution (85 mm NaCl, 75 mm sucrose, 2.5 mm KCl, 25 mm glucose, 1.25 mm NaH_2_PO_4_, 4 mm MgCl_2_, 0.5 mm CaCl_2_, and 24 mm NaHCO_3_) in an orientation optimized to preserve the mossy fiber tract in the hippocampal CA3 area (Bischofberger et al., 2006). The slice was incubated at 32°C for 60 min after sectioning, and was stored at room temperature until the experiment. The recording solution was composed of 126 mm NaCl, 2.5 mm KCl, 26 mm NaHCO_3_, 2 mm CaCl_2_, 2 mm MgCl_2_, 1.25 mm NaH_2_PO_4_, and 10 mm glucose (equilibrated with 95% O_2_ and 5% CO_2_ gas mixture). The patch pipette was pulled from borosilicate glass capillary (inner diameter: 0.75 mm, outer diameter: 1.5 mm; Sutter Inc.). The pipette was filled with an intracellular solution containing 90 mm K-gluconate, 43.5 mm KCl, 1.8 mm NaCl, 1.7 mm MgCl_2_, 0.05 mm EGTA, 10 mm HEPES, 2 mm Mg-ATP, 0.4 mm Na_2_-GTP, 10 mm phosphocreatine, 8 mm biocytin, and 20 μm Alexa Fluor 594 hydrazide (pH 7.25). The recordings were performed at 35°C. For patching, axon was visualized with an upright IR-DIC microscope (Eclipse FN-1; Nikon) equipped with high-numeric aperture objective (Nikon 1.1 NA, Apo LWD 25 W), oil condenser (Nikon D-CUO DIC Oil Condenser, 1.4 NA) and a sCMOS camera (Andor Zyla 5.5). After the recordings the Alexa Fluor 594-labeled structure was visualized *in situ* using a confocal system (Nikon Eclipse C1 Plus). Then, the slice was fixed for further morphologic experiments (see below).

To record from the individual small MF terminal, we searched for visually identifiable axonal structures under the guidance of the DIC optics in the stratum lucidum of the CA3 area, whose size was smaller than those of the typical large mossy fiber boutons. After the seal formation, we manually carefully compensated the C_pip_ in VC mode. Compensated capacitive responses to −20-mV step command were then recorded and the average of 164 sweeps served as target for tuning the C_pip_ in the model. After establishing the whole-cell configuration by applying sudden negative pressure, we switched to CC mode to record the passive and active membrane responses from the axon with different CPN compensation. All recordings were collected with a MultiClamp 700B amplifier (Molecular Devices) without filtering (filter bypassed) and digitized with Digidata1440 A/D board (Molecular Devices) at 250-kHz sampling rate using the pClamp10 software package (Molecular Devices). At the end of the experiment, we collected preliminary morphologic data by imaging the Alexa Fluor 594 signal with the confocal system. The obtained z stack image was then used to confirm the MF identity (evidenced by the presence of a large MF terminal along the recorded axon) and for the documentation of the recording position.

### 3D morphologic reconstruction

After the recording, slice was fixed overnight at 4°C in 0.1 mm phosphate buffer containing 2% PFA and 0.1% picric acid. The slice was then re-sectioned (to 60 μm) and the sections were incubated overnight with Alexa Fluor 594-conjugated streptavidin in 0.5% Triton X-100 and 2% normal horse serum to reveal the biocytin signal. To investigate the detailed morphology, the recorded axon was imaged with a confocal system (60× objective, Plan Apo VC, NA = 1.45, Nikon C2, *x-y* pixel size: 0.08–0.1 μm, z-step: 0.1–0.15 μm). High-resolution reconstructions were done automatically by the Vaa3D software (Peng et al., 2010, 2014a,b).

### Implementation of the seal in the model

The seal was represented as a single resistor (R_seal_) connected in parallel to the cell. To estimate the R_seal_ in the axonal recording, first we calculated the ratio (R_ratio_) between the cellular input resistance (R_cell_) and the R_seal_ based on the voltage shift produced by the shunt conductance of seal (Perkins, 2006):

$${\text{V}}_{\text{measured}}/{\text{V}}_{\text{rest}}={\text{R}}_{\text{ratio}}/(1+{\text{R}}_{\text{ratio}}),$$

where V_measured_ is the recorded voltage, V_rest_ is the native resting membrane potential. R_seal_ and R_cell_ were then calculated from the measured input resistance (R_in,measured_) and from the R_ratio_ using the equations:

$$1/{\text{R}}_{\text{cell}}=1/{\text{R}}_{\text{in},\text{measured}}-1/{\text{R}}_{\text{seal}},$$

where:

$${\text{R}}_{\text{seal}}={\text{ratio*R}}_{\text{cell}}.$$

The measured resting membrane potential of the recorded MF axon was −75.2 mV, close to the resting membrane potential previously reported for the somata and axons of granule cells (∼−80 mV; Staley et al., 1992; Schmidt-Hieber et al., 2004; Ruiz et al., 2010; Brunner and Szabadics, 2016; Martinello et al., 2019). This moderate shift, caused by the leak through the seal, suggests orders of magnitude larger R_seal_ compared with the axonal input resistance. Indeed, we calculated 65.64 GΩ R_seal_ and 3.94-GΩ R_cell_ for the recording assuming −80 mV resting membrane potential. Accordingly, we applied 65.64-GΩ R_seal_ in the AP reconstitution model (Figs. 4-6), while R_seal_ was set to 50 GΩ in all other simulations. To compensate for the seal-induced depolarizing voltage shift, baseline membrane potential in the model was adjusted by constant current injection to match those of the experimental data.

### General simulation parameters

Simulations were run on a custom workstation computer (i7-8700K CPU at 3.7 GHz) under Windows 10 Pro operating system, except for the AP fits. The parallelized codes for AP reconstitution were run on the Comet supercomputer through the Neuroscience Gateway portal (Sivagnanam et al., 2013). Cellular membrane parameters were assumed to be spatially uniform, unless stated otherwise. Potassium and sodium equilibrium potentials were set to −77 and +70 mV, respectively, and we assumed −80-mV reversal potential for the leak conductance in active models. Simulations ran with 4- and 0.5-μs temporal resolution, in CC mode and in VC mode, respectively. The instrumental and cellular parameter values used in different model configurations are summarized in Table 1.

**Table 1:** List of instrumental and neuronal parameters used in the different model configurations

	Total instrumental capacitance	Access resistance	Seal resistance	Capacitance neutralization	Bridge balance	Morphologic representation	Specific membrane capacitance	Internal resistivity	Specific membrane resistivity	Maximal Na^+^ conductance	Maximal K^+^ conductance
Symbol (unit)	C_tot_ (pF)	R_access_ (MΩ)	R_seal_ (GΩ)	CPN (pF)	BB (MΩ)		C_m_ (μF/cm^2^)	R_i_ (Ω*cm)	R_m_ (kΩ/cm^2^)	g_max_Na (mS/cm^2^)	g_max_K (mS/cm^2^)
AP reconstitution/native AP rediction (Figs. 4-6)	7.097	53.2	65.64	6.5/7/7.386	185	Detailed anatomic reconstruction	0.65	147.3	60.01	159.02/151.7/ 160.03^*1^	5.83/5.41/ 6.32^*1^
Correlations between signal distortions and recording conditions (Fig. 7)	6.74	1–200	50/5^*2^	6.8/ 6.3	1–200	Single isopotential compartment (100 cm^2^/1000 cm^2^ surface area)	1	1e-9	50	150	40
Correlations between signal distortions and axonal morphology (Fig. 8)	6.74	60	50	6.8	60	Detailed anatomic reconstruction	1	150	50	300	15
Testing reliability of C_tot_ estimation (Extended Data Fig. 4-1*B*)	5.8–13.7	n/a.^*3^	50	n/a.^*3^	n/a.^*3^	n/a.^*3^	n/a.^*3^	n/a.^*3^	n/a.^*3^	n/a.^*3^	n/a.^*3^
Testing reliability of cellular passive parameter and R_access_ prediction (Extended Data Fig. 4-1*C*)	6.74	60	50	6.8	60	Axon-like realistic morphology (length = 300 μm, mean diameter = 0.6 μm)	0.5–1.5	50–250	10–100	n/a.	n/a.
Testing reliability of the prediction of native AP shapes (Extended Data Fig. 4-1*D,E*)	6.74	100	50	6.8	100	Single compartment (length, diameter = 15 μm)	1	150	10	174^*4^	21^*4^

^*1^mean of the predicted maximal conductance densities for the reconstruction of APs recorded with CPN = 6.5, 7, and 7.386 pF.

^*2^for the small and for the larger cell, respectively.

^*3^not applicable in the simulated on-cell VC recording configuration.

^*4^median of the predicted maximal conductance densities for the reconstruction of APs generated with independent conductance mechanisms.

### Fitting procedures

We fitted the model responses to the experimental data to minimize the sum of squared error between them using the Brent’s PRAXIS optimization algorithm embedded in NEURON. For C_pip_ estimation, a 5-ms-long trace was considered with 2.5-ms baseline before the stimulus. For passive parameter estimations, we weighted the voltage response evoked by short current stimulus (25.8 ms from the stimulus onset, 3×) to equalize the contribution of short and long pulse responses to the total error. For AP reconstruction, fitting interval started with 2.6-ms-long baseline period before the stimulus onset. The actual APs were weighted 8-fold, starting 0.5 ms before the peak. The optimization ended 1.5 ms following the AP peak to avoid the contamination of the parameter estimation with afterdepolarization related mechanisms not implicated in the model (Ohura and Kamiya, 2018; Martinello et al., 2019).

We used sodium and potassium conductance mechanisms with canonical Hodgkin–Huxley gating scheme, in which not only the density can be freely adjusted but also the kinetics and the voltage dependence of the two types of conductance. For this, we modified the built-in Hodgkin–Huxley mechanisms of NEURON by introducing rate-scaling factors (scNa and scK) to adjust the speed of model channel operation. The originally implemented temperature scaling was removed from the mechanisms. In addition, we used global voltage shifts (vsNa and vsK) to modulate the voltage dependence of the channels. Altogether, free parameters (g_Na_max, g_K_max, scNa, scK, vsNa, vsK) were constrained to obtain an ideal model of the AP waveform measured in our various experimental settings. Because the R_access_ can change during the recordings, this parameter was set individually for each target trace. Optimization of the individual AP fits was initiated from four parameter sets that allows exploration of substantial part of the parameter space (Extended Data Fig. 4-2). To assess the quality of individual fits, their mean squared error was normalized to the baseline variance of the actual target trace. Of the results of the four parallel optimizations, the solution where the normalized error was the smallest was accepted as the best fit. Fit was rejected when the normalized error was larger than 10. Fitting of two of the original 90 target APs did not resulted in solutions, which met this criterion. These APs were excluded from the analysis (both were obtained with 7.386 pF CPN).

### Assessments of the reliability of optimizations

*In silico* reconstruction of the complete experiment consisted of subsequent optimization steps (Extended Data Fig. 4-1*A*). First, we used VC data to set actual pipette parameters (1). After adding the model instrumentation and the detailed morphology of the recorded structure to the model, we tuned the passive cellular parameters together with the R_access_ (2). Next, we equipped the model structure with active sodium and potassium conductances and adjusted their properties to match model responses the experimentally recorded AP waveform (3). Finally, having established the appropriate conductance sets, we obtained the native behavior of the axon (4). The reliability of the fitting procedures was tested with artificial traces with Gaussian noise.

To assess the accuracy of C_tot_ estimation, we generated target traces by simulating on-cell VC experiments. The model always recovered correct C_tot_ value (proportion of fitting estimations within 10% error to the correct value: 100%, *n* = 990 optimizations; Extended Data Fig. 4-1*A*,*B*) regardless to the C_tot_ (5.8–13.7 pF) and noise level (SD = 0–35.3 pA) of the targets, which confirms the high sensitivity of VC based C_pip_ estimation.

To test the reliability of cellular passive parameter and R_access_ prediction, we created a hypothetical axon with biologically plausible diameter distribution (log-normal distribution with mean of 0.6 μm and variance of 0.4 μm^2^). We attached the model axon to the pipette and generated hypothetical CC measurements with long and short current stimuli (C_m_ range: 0.5–1.5 μF/cm^2^, R_m_ range: 10–100 kΩ/cm^2^, R_i_ range: 50–250 Ω*cm, R_access_: 50–400 MΩ, noise SD = 1.73 mV). We fitted these synthetic targets from a single initiation parameter set (C_m_: 1 μF/cm^2^, R_m_: 50 kΩ/cm^2^, R_i_: 150 Ω*cm, R_access_: 150 MΩ). 75% of optimizations resulted in acceptable results, that is, the fit error was <10-times the baseline variance of its target. In those successfully fitted cases the predicted parameters were close to their predefined values (proportion of fitting estimations within 10% error to the correct value: C_m_: 90.83%, R_m_: 97.66%, R_i_: 81.88%, R_access_: 82.3% *n* = 469 optimizations; Extended Data Fig. 4-1*C*).

We also verified the reliability of the prediction of native AP shapes in independent simulations (Extended Data Fig. 4-1*D*,*E*). For this we generated AP waveforms using independent, sophisticated sodium and potassium conductance mechanisms using 8-state kinetic schemes obtained from (Schmidt-Hieber and Bischofberger, 2010; Hallermann et al., 2012) in a single compartment neuron (C_m_: 1 μF/cm^2^, R_m_: 10 kΩ/cm^2^, R_i_: 150 Ω*cm, diameter of the compartment: 15 μm). Channel densities and kinetics were systematically varied (g_Na8st_max range: 150–450 mS/cm^2^, g_K8st_max range: 250–850 mS/cm^2^, channel kinetics were scaled by a factor of 2–4) to generate diverse AP shapes (AP peak range: 19.28–65.82 mV, AP half-width range: 0.21–0.62 ms). We next simulated CC measurements with these conductance sets to generate noisy targets (R_access_ = 100 MΩ, C_pip_ = 6.74 pF, BB compensation = 100 MΩ, CPN compensation = 6.8 pF, R_seal_ = 50 GΩ, added noise SD: 0.45 mV) for subsequent optimization. Our standard fitting routine reliably retrieved the native AP shapes.

### Systematic errors in model predictions

We also examined the potential impact of systematic error sources on model predictions. Such potential error can originate from inaccuracies of the morphologic reconstruction. *Post hoc* anatomic processing can result in considerable tissue shrinkage. Additionally, diameter of thin axonal processes is close to the resolution limit of light microscopy. To evaluate the potential effects of inaccurate anatomic representation of the recorded axon caused by tissue shrinkage or imperfect optical resolution, we artificially altered the reconstructed morphologies and tested how the recovered native APs were affected. For testing the impact of inaccurate diameter estimation, axonal diameters were either homogeneously increased by 160 nm or reduced by 180 nm (axons were not allowed to shrink below 100 nm). In another control simulation we proportionally increased the axonal diameters by 30% to compensate for potential tissue shrinkage. As expected, recovered passive parameters and active conductance densities scaled with the diameter to compensate for the altered morphologic dimensions (Table 2). However, as the model re-adjusted the local electrical environment by scaling the membrane properties, the predicted intra-axonal and native AP waveforms remained remarkably similar despite the large changes in the morphology and passive membrane parameter.

**Table 2:** Effect of axonal diameter and the R_seal_ on the model predictions

		Standard model	Reduced R_seal_	Reduced diameter (−180 nm)	Increased diameter (+160 nm)	Increased diameter (+30%)
Passive parameters	R_m_ (kΩ/cm^2^)	60.01	72.92	38.88	62.97	66.83
	C_m_ (μF/cm^2^)	0.65	0.62	0.99	0.62	0.58
	R_i_ (Ω*cm)	147.3	141.54	56.78	208.08	205.03
	Normalized error	2.96	3.05	3.18	2.95	2.93
Active conductances for APs recorded witd CPN = 6.5 pF	gNa (mS/cm^2^)	159.02 ± 1.73	160.24 ± 2.1	229.79 ± 3.57	150.24 ± 1.86	146.63 ± 1.55
	gK (mS/cm^2^)	5.83 ± 0.05	5.46 ± 0.04	12.74 ± 0.56	5.33 ± 0.04	5.29 ± 0.05
	Normalized error	5.2 ± 0.16	5.09 ± 0.16	5.72 ± 0.19	5.22 ± 0.16	5.35 ± 0.17
Active conductances for APs recorded witd CPN = 7 pF	gNa (mS/cm^2^)	151.7 ± 0.95	150.29 ± 1.06	232.6 ± 1.5	142.27 ± 0.97	137.95 ± 0.85
	gK (mS/cm^2^)	5.41 ± 0.03	5.08 ± 0.03	9.23 ± 0.08	5.08 ± 0.03	4.85 ± 0.03
	Normalized error	5.89 ± 0.17	5.92 ± 0.17	6.04 ± 0.19	6.36 ± 0.18	6.09 ± 0.17
Active conductances for APs recorded witd CPN = 7.386 pF	gNa (mS/cm^2^)	160.03 ± 1.47	142.26 ± 1.17	249.13 ± 2.44	144.38 ± 1.23	138.84 ± 1.08
	gK (mS/cm^2^)	6.32 ± 0.09	6.08 ± 0.44	11.24 ± 0.146	5.87 ± 0.07	5.71 ± 0.05
	Normalized error	6.8 ± 0.28	8.22 ± 0.34	7.35 ± 0.3	8.1 ± 0.3	7.49 ± 0.26
*n = 30 AP reconstitution*						
AP parameters (reconstructed measurement)	dV/dt_max_ (V/s)	363.26 ± 0.93	369.87 ± 6.04	372.7 ± 0.96	354.99 ± 0.8	364.5 ± 0.78
	Peak (mV)	9.01 ± 0.12	9.01 ± 0.49	9.24 ± 0.12	8.98 ± 0.12	8.93 ± 0.12
	Threshold (mV)	−45.28 ± 0.07	−45.29 ± 0.06	−45.69 ± 0.06	−45.17 ± 0.07	−45.36 ± 0.06
	Half-width (ms)	0.52 ± 0.002	0.52 ± 0.004	0.52 ± 0.002	0.51 ± 0.002	0.52 ± 0.002
AP parameters (local in the axon)	dV/dt_max_ (V/s)	453.11 ± 2.54	453.82 ± 2.53	481.59 ± 3.06	450.44 ± 2.34	459.79 ± 2.34
	Peak (mV)	21.96 ± 0.12	21.23 ± 0.12	22.34 ± 0.11	22.13 ± 0.12	22.08 ± 0.12
	Threshold (mV)	−35.88 ± 0.09	−35.92 ± 0.09	−36.97 ± 0.09	−36.01 ± 0.09	−36.12 ± 0.09
	Half-width (ms)	0.48 ± 0.002	0.49 ± 0.002	0.49 ± 0.002	0.48 ± 0.002	0.48 ± 0.002
AP parameters (native, I-step evoked)	dV/dt_max_ (V/s)	1064.81 ± 5.1	1111.03 ± 5.76	914.95 ± 6.32	865.03 ± 4.65	854.73 ± 4.62
	Peak (mV)	46.85 ± 0.09	47.34 ± 0.09	40.26 ± 0.15	40.06 ± 0.12	38.67 ± 0.14
	Threshold (mV)	−37.36 ± 0.09	−37.64 ± 0.09	−36.7 ± 0.09	−35.93 ± 0.09	−35.67 ± 0.09
	Half-width (ms)	0.33 ± 0.002	0.33 ± 0.002	0.34 ± 0.002	0.33 ± 0.002	0.34 ± 0.002
AP parameters (native, propagating)	dV/dt_max_ (V/s)	1605.96 ± 9.75	1671.34 ± 10.82	1549.38 ± 12.2	1609.7 ± 10.18	1630.3 ± 9.82
	Peak (mV)	64.21 ± 0.07	64.71 ± 0.07	63.29 ± 0.11	64.16 ± 0.08	64.24 ± 0.07
	AP width at −10 mV (ms)	0.36 ± 0.002	0.35 ± 0.002	0.36 ± 0.002	0.36 ± 0.002	0.36 ± 0.002
	Propagation velocity (ms)	0.45 ± 0.001	0.48 ± 0.002	0.43 ± 0.002	0.46 ± 0.002	0.46 ± 0.001

R_seal_ provides an additional error source because its calculation depends on the native resting membrane potential, which cannot be assessed directly (i.e., measurements start in cell attached mode). Therefore, we defined the theoretical lower limit for the R_seal_ in the direct axonal recording; when native resting membrane potential equals with the potassium equilibrium potential (−93 mV with the solutions used in the experiment) R_seal_ would be only 20.58 GΩ. Re-optimization of the model using this small R_seal_ caused only minor alterations in the predicted conductances and AP parameters (reduced R_seal_ model; Table 2). Altogether, these control simulations suggest that potential systematic errors have marginal impact on the primary results of the manuscript.

### Simulations to explore the correlations between signal distortions and recording conditions (Fig. 7)

We used a single-compartment neuron in these simulations (C_m_: 1 μF/cm^2^, R_m_: 20 kΩ/cm^2^, g_Na_max: 150 mS/cm^2^, g_K_max: 40 mS/cm^2^). Surface area of the compartment was set to 100 μm^2^ or 1000 μm^2^, to model a small neuronal structure with 1-pF total capacitance or a larger cell with 10-pF total capacitance, respectively. To keep the seal-induced signal distortion constant in the two conditions, R_seal_ was reduced to 5 GΩ, for the large cell, which results in similar R_seal_/R_cell_ ratio as for the small structure. The total instrumental capacitance was fixed to 6.74 pF, while R_access_ was systematically varied in the range of 1–200 MΩ. To mimic optimal recording configuration, the applied CPN settings (6.8 pF) closely matched with C_tot_. APs were elicited with 3-ms-long current stimuli (30 and 160 pA for small and larger cell, respectively).

### Simulations to explore the correlations between signal distortions and axonal morphology (Fig. 8)

The detailed morphology of a somatically labeled granule cell was imported to the NEURON. Dendritic spines were implemented by scaling 2-fold the C_m_ and the leak conductance in dendrites. Passive parameters (R_i_: 150 Ω*cm, C_m_: 1 μF/cm^2^, R_m_: 50 kΩ/cm^2^) were constant otherwise. Active conductances (g_Na_ density = 300 mS/cm^2^, g_K_ density = 15 mS/cm^2^) were homogenously distributed along the cell, except the initial part of the axon where we applied higher channel densities (g_Na_ density = 1200 mS/cm^2^, g_K_ density = 75 mS/cm^2^) with left-shifted activation and inactivation (10 mV in the hyperpolarized direction). To explore the distortions in axonal AP recordings, position of recording electrode (C_tot_ = 6.74 pF, R_access_ = 60 MΩ, R_seal_ = 50 GΩ, applied CPN = 6.8 pF) was systematically changed along the main axon (*n* = 942 recording positions). APs were evoked by 3-ms-long current stimuli at each recording position. Amplitude of the injected current was automatically adjusted to evoke APs ∼1.5 ms (1.33 ± 0.01 ms delay, *n* = 942 APs) after stimulus onset. Local capacitance was determined for each recording position using idealized VC simulations in the absence of experimental instrumentation (built-in SEClamp mechanism with 10-MΩ series resistance). For quantification, we integrated the first 100 μs of the transient capacitive response to −20-mV voltage step. The resulted charge was divided by the voltage step amplitude.

### Data analysis and statistics

AP threshold was determined as membrane voltage where depolarization rate exceeded 20 mV/ms. AP amplitude was calculated as the voltage difference between the absolute peak potential and the threshold. AP half-width was defined as the spike duration at half of its amplitude. AP conduction velocity was determined by measuring the temporal difference between AP peaks at the initiation and at the recording site.

Data were analyzed using pClamp (Molecular Devices), OriginPro (OriginLab), Excel (Microsoft), Photoshop (Adobe), and custom written NEURON or Python scripts. Voltage values are presented without correction for the liquid junction potential. Normality of the data were assessed with Shapiro–Wilk test. Population data are presented as mean ± SEM.

### Code accessibility

The relevant NEURON codes described in the paper are freely available online at https://github.com/brunnerjanos/amplifier-model or can be downloaded from the ModelDB (http://modeldb.yale.edu; accession number: 267063). This NEURON codes are also available as Extended Data 1.

10.1523/ENEURO.0059-21.2021.ed1 Extended Data 1 NEURON codes producing data shown in Figures 2, 5, 8. Download Extended Data 1, ZIP file.

## Results

### *In silico* implementation of amplifier features

We developed and validated a realistic amplifier model working both in voltage clamp (VC) and in current clamp (CC) mode using the NEURON simulation environment (Hines and Carnevale, 1997). The model needed to be suitable for both VC and CC modes, because instrumental compensations are typically determined in VC mode during seal formations and these settings are implemented for CC during voltage signals recordings. For precise implementation of the components of the amplifier circuit, we examined their capacitive and resistive properties using three test configurations (Extended Data Fig. 1-1). The first test configuration was the isolated headstage in open circuit (test #1), which allowed the characterization of the high-frequency boost unit (see below) and the capacitance compensation of the VC circuitry. Since open circuit measurements are not possible in CC mode, the circuit was closed via known resistors in the second test configuration (test #2). This configuration allowed the estimation of stray capacitance associated with the feedback resistor. The third test circuit was a modified model cell (test #3, type 1U, Molecular Devices), which represents whole-cell recording conditions as it includes an idealized cell and recording pipette with their resistive and capacitive components. In order to explore an extended range of signal processing capacity of the amplifier, model cell components were varied using custom capacitors and resistors. This test configuration allowed us to investigate the features of the CPN of the CC circuitry.

We used idealized circuits for model amplifier construction to start with, namely, a resistive feedback circuit (current-to-voltage converter) for the VC and a voltage follower circuit (a unity gain voltage buffer) with idealized current source for the CC (Wilson and Park, 1989; Sigworth, 1995; Sherman-Gold, 2012; Fig. 1). Operation of combined VC and CC amplifiers requires a capacitor (C_inj_) for current injection in both VC and CC modes and a resistor (R_f_) used as current generator in CC and as feedback resistor in VC (Strickholm, 1995; Fig. 1). To create realistic amplifier model, we first measured amplifier responses using the three test circuits, which were subsequently simulated allowing individual tuning of elementary circuit parameters. First, we measured C_inj_, assuming that the total capacitive load present in the isolated VC circuit (test #1) corresponds to the injector capacitor. By recording voltage-step-evoked capacitive current responses in open circuit configuration, we determined that the total capacitive load is 1.615 pF in our amplifier (MultiClamp700B). As virtually all real resistors, R_f_ has a certain amount of parasitic capacitance (C_stray_) which affects the performance characteristics of the amplifier. We next determined the size of this parasitic capacitance. In CC mode, the C_stray_ connected in parallel with the R_f_ (Fig. 1, ISOURCE circuit) acts as a capacitance-neutralizing element. However, this neutralizing effect appears only when the input load is considerably smaller than the R_f_ which was set to 500 MΩ in the real amplifier (Fig. 2*A*, left traces). To characterize the size of C_stray_ associated with the R_f_, we adjusted the C_stray_ in the model to reproduce the evoked voltage responses recorded with the amplifier in test #2 configuration with 20-, 50-, and 100-MΩ input loads (Fig. 2*A*, right traces). These simulations revealed 0.38 pF C_stray_, thus we used this value in the model.

Next, we added the remaining amplifier components to the model one-by-one. First, we focused on the VC operations whose speed depends on a dedicated high-frequency boost circuit (Sigworth, 1995). Amplifiers are built with this compensation mechanism because C_stray_ substantially reduces the output bandwidth of the feedback circuit in VC mode (time constant of the capacitive relaxation with the previously determined 0.38 pF C_stray_: 191.92 μs; Fig. 2*B*, left). We added a simplified boosting unit tuned to accelerate model responses to the experimentally observed amplifier speed (3.85 vs 3.19 μs, real vs boosted model; Figs. 1, 2*B*, right). Next, we implemented two pipette capacitance compensation circuits in the VC model. Fast capacitance compensation (CPf, 0–16 pF, 0.5–1.8 μs) cancels the majority of C_pip_-induced current transients, while the slow capacitance compensation (CPs, 0–3 pF, 10–4000 μs) reduces the slower instrumental capacitive components (Sigworth, 1995; not equivalent to whole-cell compensation that was not implemented in this model; Figs. 1, 2*C*).

We also implemented two compensatory mechanisms for the CC mode, namely the CPN and the BB compensation (Fig. 1). The CPN circuit is a positive feedback loop that feeds the pipette voltage back to the input through the C_inj_ to discharge the pipette capacitance (Wilson and Park, 1989; Sherman-Gold, 2012). Because of this positive feedback, the recordings are prone to oscillate as CPN level approaches a fully compensated state. Such evolving oscillation carry information about the intrinsic behavior of the CPN circuit. Therefore, the oscillating signal (dampening and frequency profile) can be employed for determining a minimal set of passive circuit elements necessary the re-create the CPN behavior. Specifically, we measured the maximal possible CPN where the recording is still stable using test #3 circuit. Pipette parameters (10 MΩ, 2.8 pF) and the cell-equivalent resistor (500 MΩ) was fixed and only the capacitance of the cell was varied from 0.75 to 46.7 pF. The model reproduced the recorded signal artefacts when a resistor (1.49 MΩ) and an inductor (18.3 H) was incorporated to the CPN circuit in series with the C_inj_ (Figs. 1, 2*D*). Amplifiers are typically supplied with BB compensatory mechanism to eliminate the voltage drop across the access resistance (Araki and Otani, 1955; Sherman-Gold, 2012). In the model, we subtracted a scaled version of the command signal from the recorded voltage (Fig. 1). We also extended our model with a four-pole low-pass Bessel filter unit with adjustable cutoff frequencies from 0.5 to 100 kHz (Fig. 1).

Altogether, by using a measurement-based approach we created an amplifier model, in which both the VC and CC operation and their specific compensatory capabilities show realistic behavior.

### Pipette implementation considers the observed nonuniform C_pip_ and R_pip_ distributions

**Figure 3.**
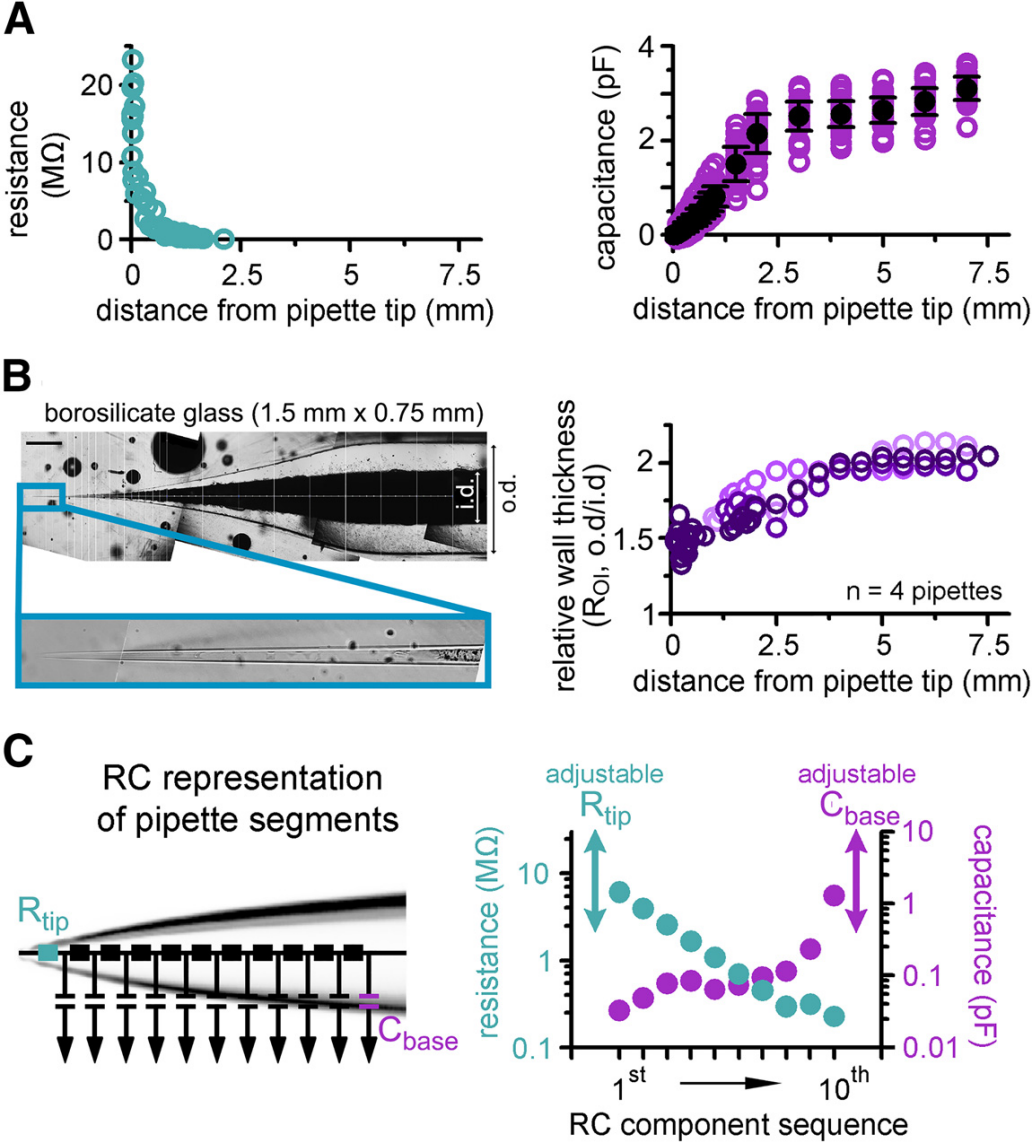
Implementation of pipettes with non-uniform C_pip_ and R_pip_ distributions. ***A***, Pooled data of R_pip_ (left graph) and C_pip_ (right graph) as a function of tip distance. ***B***, A representative imaging plane that was used for the measurement of the relative wall-thickness (R_OI_) that is, the ratio of outer and inner diameter (o.d. and i.d., respectively) of the pipettes. Scale bar: 0.5 mm. Graph on the right shows that the wall of the pipettes is much thinner toward the tips (i.e., the i.d. is larger than predicted from the R_oi_ of the original glass; the four measured pipettes are shown in different shades of purple). ***C***, Pipettes were implemented as 10 independent RC units, a resistor (R_tip_, on the left) and a capacitor (C_base_, on the right). The two latter components allow the adjustment of the model to fit the differences of R_pip_ and C_pip_ of individual pipettes. Graph on the right indicates the actual capacitance (purple) and resistance (green) values of the 10 fixed RC motifs.

Next, we focused on the accurate implementation of patch pipettes. To properly characterize the distribution of resistance along patch pipettes that are suitable for recordings from small axons, we repeatedly broke small pieces from the end of the pipettes and determined the resistance as a function of tip distance (*n* = 55 resistance measurements; Fig. 3*A*). In agreement with the theoretical considerations (Benndorf, 1995; Ying et al., 2002), we found that pipette resistance drops sharply after the tip, falling below 1 MΩ within the first millimeter (R_pip_ declines from the tip with length constant of 208 ± 40 μm and fall below 1 MΩ at 703-μm tip distance, exponential fit of the data shown in Fig. 3*A*; *R*^2^ = 0.86, *n* = 55 resistance measurements; R_pip_ = 0.33 ± 0.18 MΩ for measurements with tip distance over 1 mm, *n* = 20 measurements). To determine the capacitance distribution of our pipettes we systematically varied the immersed length of the pipettes in the recording solution and measured the capacitance, which corresponds to the cumulative capacitance of the dipped part (Cornwall and Thomas, 1981; *n* = 656 capacitance measurements; Fig. 3*A*). The cumulative capacitance increased within the first 3 mm from the tip, whereas the remaining part of the pipette had only moderate contribution to the total capacitance suggesting inhomogeneous capacitance distribution along pipettes (0.03 ± 0.01, 0.48 ± 0.02, 2.52 ± 0.05, and 3.1 ± 0.04 pF at 0.1-, 0.7-, 3-, and 7-mm tip distances, respectively, *n* = 39, 39, 40, and 38 measurements; Fig. 3*A*). A key parameter that defines the pipette capacitance is the ratio of the outer and inner pipette diameters (R_OI_; Cornwall and Thomas, 1981; Benndorf, 1995). One potential explanation for the inhomogeneous capacitance distribution is that the R_OI_ is not constant along the pipette but it decreases toward the tip. To test this possibility and verify the predictions of the capacitance distribution measurement, we directly measured the R_OI_ of the recording pipettes. In order to precisely measure the edge of the pipette walls and avoid optical distortions by the curved glass walls, we carefully split the pipettes using a custom-built grinding system and measured their inner and outer diameters along the longitudinal axis (Fig. 3*B*; for details, see Materials and Methods). Consistent with the dipping measurements, R_OI_ decreased toward the tip (R_OI_ <2 mm = 1.6 ± 0.02 vs R_OI_ >5 mm = 2.04 ± 0.02, *n* = 4 pipettes) explaining the larger contribution of the tip region to the total pipette capacitance.

Based on these measurements, we created the skeleton of a “prototypical” patch pipette model from 10 RC units to consider the inhomogeneous distribution of capacitance and resistance (Fig. 3*C*). Model pipette parameters (R_pip_ and C_pip_) can be adjusted using an additional resistor and capacitor to account for variability across individual pipettes. Since the bulk of pipette capacitance is spatially separated from the pipette resistance and because these pipettes have similar a shape, we placed the tunable resistor to the tip and the capacitor to the back of the pipette model (R_tip_ and C_base_; Fig. 3*C*).

### Reconstitution of the native electrical behavior of a small axon

**Figure 4.**
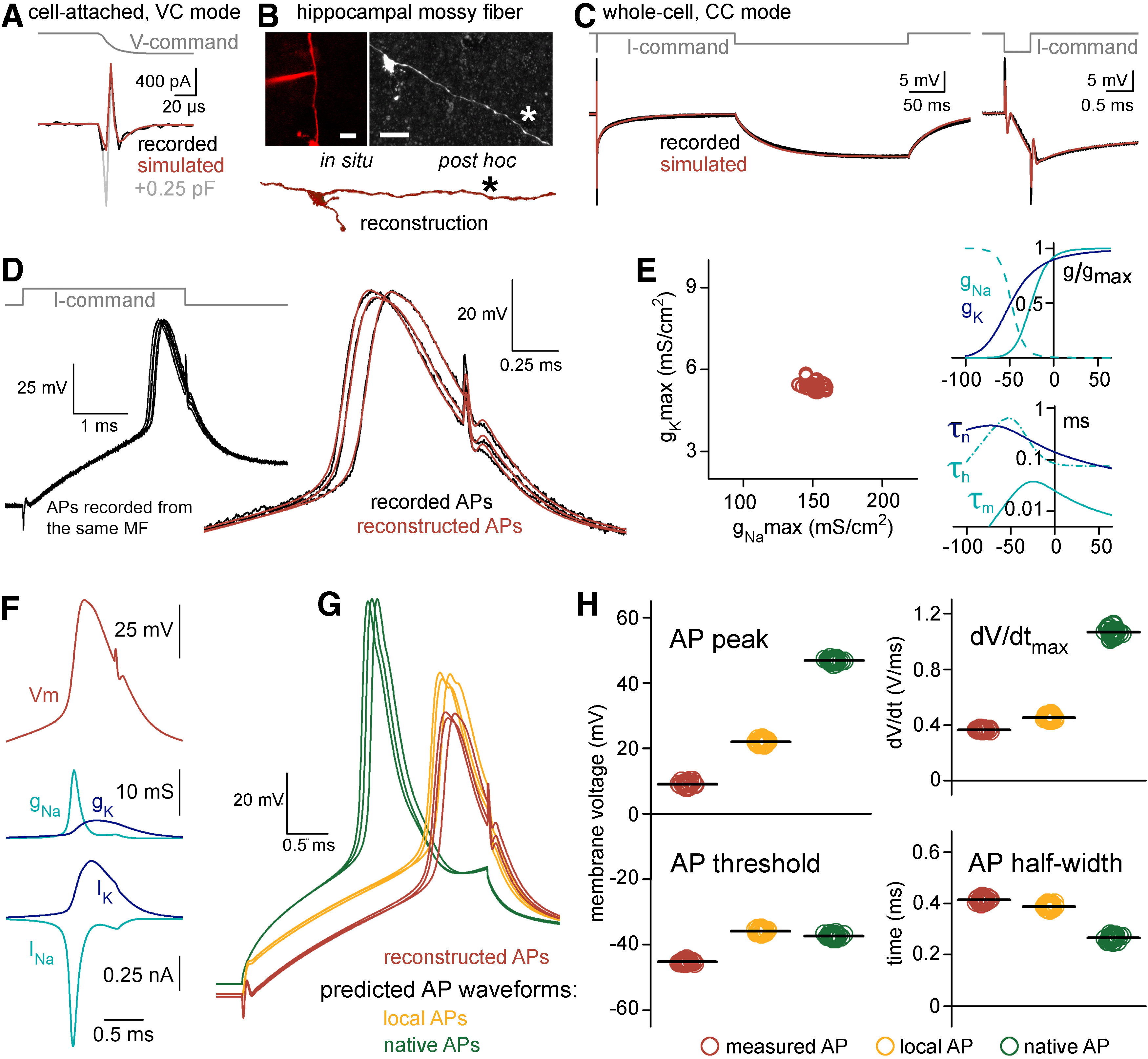
Reconstitution of the undisturbed membrane dynamics of a recorded axon. ***A***, Current responses to −20-mV voltage steps recorded from the axonal membrane in on-cell mode (black) and in the model with 7.097 pF (red) and 7.347 pF (gray) C_tot_. Notice the sensitivity of simulated responses to small differences in the instrumental capacitance applied in the model. ***B***, Confocal z-stack images show the recorded axon at the end of the experiment (left) and after the anatomic recovery (right). Bottom, Part of the reconstructed morphology. Asterisk marks the recording position. Scale bars: 10 μm. ***C***, Voltage responses in the recorded axon (black) and in the passive cable model with the added instrument (red) to short (3 ms, −50 pA) and long (250 ms, −2.5 pA) current stimuli. The short pulse is shown at an expanded timescale on the right. ***D***, Recorded (black) and simulated APs (red) evoked by brief (3 ms, 86 pA) current stimuli. APs were simulated in the complex model that included the instrument, the passive cable and Hodgkin–Huxley type sodium and potassium conductances. To evaluate the reliability of the optimization, see Extended Data Figures 4-1, 4-2. ***E***, left, Distribution of optimal maximal Na^+^ (g_Na_max) and K^+^ (g_K_max) conductance densities obtained from 30 recorded and independently fitted APs. Right, Averaged gating properties and voltage dependence of the conductances that were resulted in by the fits that recreated the recorded APs. ***F***, Simulated AP waveform (top) with the underlying conductances (middle, g_Na_ and g_K_) and the modeled ionic currents (bottom, I_Na_ and I_K_). ***G***, The simulations with the confirmed conductance sets allowed to see the waveforms of the same APs not only within the pipette (red, corresponding to measured APs) but also the simultaneous AP waveforms within the axon (orange, corresponding to local APs) and, after the removal of the recording instrument from the model, the native, undisturbed AP waveforms (green). ***H***, Differences in waveform parameters of measured, local and native APs. Individual points show the peak, threshold, maximum rate of the rise (dV/dt_max_) and half-width of 30 independently simulated APs in the three points of view. Horizontal black lines indicate mean values.

To test the efficacy of the complex instrumental model in predicting undisturbed fast neuronal membrane responses from distorted recordings, we used patch-clamp data from a small (<1 μm) en passant axonal varicosity of an identified hippocampal mossy fiber axon (MF; Fig. 4*A*). Recordings from submicron-sized neuronal structures are supposedly substantially distorted as they can be patched only with high access resistance and their capacitance is in the range of the remaining uncompensated instrumental capacitance.

10.1523/ENEURO.0059-21.2021.f4-1 Extended Data Figure 4-1 Parameter extraction from recorded VC and CC data. ***A***, Outline of the consecutive optimization steps that were used to retrieve the instrumental and the cellular parameters. ***B***, Distribution of the relative error in C_pip_ estimations. ***C***, Distribution of the relative errors present in the R_access_ and passive parameter estimations. ***D***, Overlay of representative APs (native and measured, gray and black traces, respectively) generated using 8-state active conductance models and corresponding best-fit APs generated with the standard conductances used in our final model (reconstructed and predicted, red and green traces, respectively). ***E***, Comparison of measured AP half-width and peak values (black) and the corresponding predicted native parameters (green) with the original value (*n* = 90 simulated experiments). The line indicates equality. Download Figure 4-1, TIF file.

10.1523/ENEURO.0059-21.2021.f4-2 Extended Data Figure 4-2 Uniqueness of the fit results. The explored parameter space during the optimization of the conductances needed to reconstruct the recorded APs (30 fitted APs started from 120 initializations). Each dot represent single run during the fit (*n* = 142,605 runs). The optimization error of the each run is color coded. Red crosses mark best-fit solutions. Download Figure 4-2, TIF file.

The experimental conditions needed to be realistically modeled, including the precise morphology and electrical properties of the biological structure and the instrumental conditions (Extended Data Fig. 4-1; for details, see Materials and Methods). First, we characterized the total instrumental capacitance (C_tot_, the sum of all amplifier-related, holder-related, and pipette-related capacitances) present in the actual recording (C_tot_: 7.097 pF from which 3.23 pF is the C_pip_ and 2 pF is the capacitance of the pipette holder; Fig. 4*A*). Target trace for this estimation was recorded using both fast and slow pipette capacitance compensation of the amplifier with the highest frequency resolution (output filter was bypassed) and we set the model accordingly.

Next, we precisely reconstructed the morphology of the biocytin-labeled and fluorescently recovered MF axon because signal propagation strongly depends on the length, diameter and their inhomogeneties of the biological structures (Goldstein and Rall, 1974; Manor et al., 1991; Fig. 4*B*). As it is characteristic for hippocampal mossy fibers the 519-μm-long reconstruction of the recorded MF included large (>3 μm) terminals, filopodial extensions and small (<1.5 μm) en passant varicosities within the stratum lucidum of the CA3 area (Acsády et al., 1998; Rollenhagen et al., 2007).

We next determined the cable properties: specific membrane resistance (R_m_), membrane capacitance (C_m_), and intracellular resistivity (R_i_) of the particular axon. For this, we optimized the model to the experimentally recorded voltage responses evoked by short and long current stimuli (Fig. 4*C*; Roth and Häusser, 2001; Schmidt-Hieber et al., 2007; Nörenberg et al., 2010; Szoboszlay et al., 2016). The advantages of the complex (instrumental + biological) model became obvious in these fittings. Pipette artefacts can markedly contaminate the onset of the evoked responses on current injection (Major et al., 1994). The incorporation of the complete experimental instrument to the model precisely reproduced these stimulus artefacts, therefore, allowing us to isolate biological contributions and obtain the passive cellular parameters. The predicted cellular parameters (R_m_ = 60.01 kΩ/cm^2^, a C_m_ = 0.65 μF/cm^−2^, and R_i_ = 147.3 Ω*cm) match with the data reported for MFs using recordings from large terminals (Hallermann et al., 2003). It is important to note that the similarities of the simulated and recorded fast voltage transients further verify our complex model.

Next, we simulated the active ionic mechanisms underlying the recorded AP waveforms. APs were evoked with brief current stimuli (3 ms, 86 pA; Fig. 4*D*,*E*). To simulate APs we tuned Hodgkin–Huxley-type sodium and potassium conductances (Hodgkin and Huxley, 1952; modified version of the built-in mechanism in the NEURON simulation environment). Optimization of the density, kinetics and voltage dependence resulted in AP waveforms closely matching to the recorded ones (absolute AP peak: 9.01 ± 0.12 vs 9.28 ± 0.12 mV, AP half-width: 0.52 ± 0.002 vs 0.52 ± 0.002 ms, AP threshold: −45.28 ± 0.07 vs −44.27 ± 0.16 mV, maximum rate of rise: 363.26 ± 0.93 vs 355.23 ± 9.39 V/s, simulated vs recorded APs, respectively, *n* = 30 APs; Fig. 4*D*). This suggest that the complex instrumental model in combination with traditional conductance functions is sufficient to reconstruct the recorded AP waveforms from potentially distorted recordings. Importantly, although each of the target APs was fitted independently, the optimal model conductance parameters were confined to a narrow range within the parameter space (coefficient of variation = 3% for both the Na^+^ and K^+^ conductance predictions, maximal g_Na_ density: 151.7 ± 0.95 mS/cm^2^, maximal g_K_ density: 5.41 ± 0.03 mS/cm^2^, *n* = 30; Fig. 4*E*) indicating that the optimization provided a unique solution for the experimental data. Analysis of the error between the fit and its target data in different Na^+^ and K^+^ conductance combinations also revealed a single minimum that coincided with the best fit parameter combinations (Extended Data Fig. 4-2). Furthermore, the optimal conductance parameters (i.e., their maximal conductance levels, their voltage dependence, and their activation and inactivation time constants), as well as the resulted ionic currents, are similar to previously described mechanisms underlying cortical axonal APs at near-physiological temperature (Geiger and Jonas, 2000; Alle et al., 2009, 2011; Schmidt-Hieber and Bischofberger, 2010; Hallermann et al., 2012; Hu and Jonas, 2014; Engel and Jonas, 2005; Fig. 4*E*,*F*), confirming the validity of our AP-reconstitution approach.

The relatively high series resistance (R_access_) in the recording (modeled R_access_: 53.2 ± 1.02 MΩ in the AP reconstitution simulations) can result in significant pipette filtering. The complex model, however, allowed us to investigate not only the APs recorded through the pipette but also the local spike that occurred within the axon while it was patched (Fig. 4*G*). These local APs are not affected by the filtering effect caused by the pipette, so we could directly quantify filtering effects by comparing the local and recorded axonal AP waveforms (Fig. 4*H*). Because of the filtering, local spikes had larger peak amplitudes and faster time course than the recorded APs (absolute peak: 21.96 ± 0.12 vs 9.01 ± 0.12 mV, local vs measured AP, respectively, *n* = 30 APs, half-width: 0.48 ± 0.002 vs 0.52 ± 0.002 ms, local vs measured AP, respectively, *n* = 30 APs; Fig. 4*H*). As expected, the maximal rate of rise (dV/dt_max_) during the upstroke of spikes was the most different between local and measured APs as it is the most sensitive to low-pass filtering introduced by the pipette (453.11 ± 2.54 vs 363.26 ± 0.93 V/s, local vs measured AP, respectively, *n* = 30 APs; Fig. 4*H*). Altogether, these observations are in agreement with the expected filtering, which affects fast signals, such as the axonal APs more prominently.

We next investigated the native AP parameters predicted by the model. In this arrangement, we run the modeled biological structure with the reconstructed model conductances but the recording instruments was removed (Fig. 4*G*,*H*). Thus, we predict how the native APs would look if the recording instrument was not present. We found that the native APs reached more depolarized peak potential (46.85 ± 0.09 mV, *p* ≪ 0.0001, paired sample *t* test; *n* = 30 APs) and were significantly faster (half-width: 0.33 ± 0.002 ms, *p* ≪ 0.0001, paired sample *t* test; dV/dt_max_: 1064.81 ± 5.1 V/s, *p* ≪ 0.0001, paired sample *t* test, *n* = 30 APs) compared with the local spikes modeled with the experimental rig.

Altogether, these results show that it is possible to obtain the native and local AP properties and the plausible underlying mechanisms using a complex instrumental model.

### Modeling the recording instruments accurately predicts signal distortions and native AP shapes

**Figure 5.**
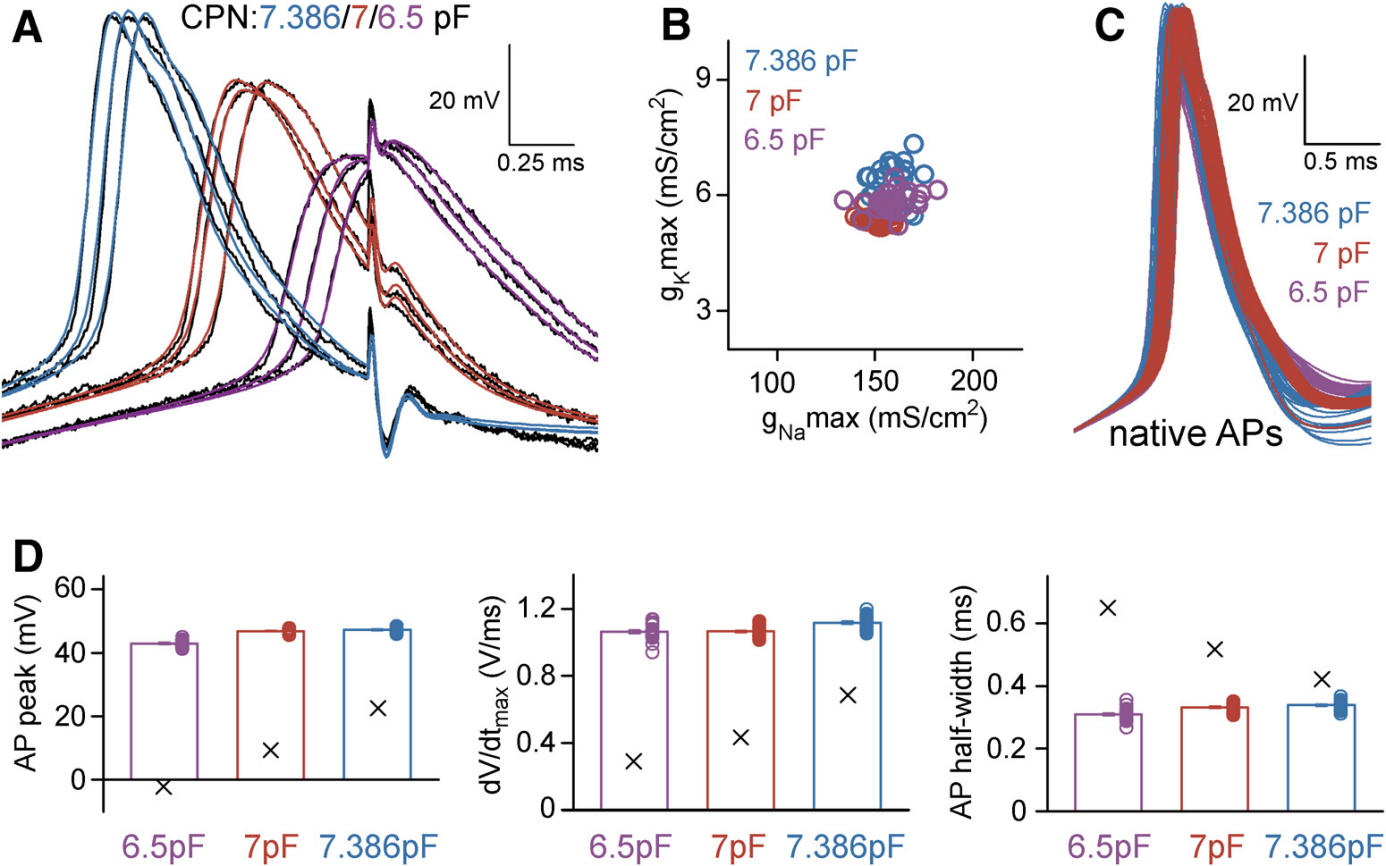
APs recorded and simulated from the same axon under different instrumental distortions predicted similar native spike shapes. ***A***, Representative target APs (black) and their best-fit representations in the complex instrument+axon model with standard CPN settings (red, 7 pF), with slightly reduced CPN (purple, 6.5 pF) or with the highest attainable CPN level (blue, 7.386 pF). The parameters of the APs recorded with different CPN settings are shown in the Extended Data Figure 5-1. ***B***, The best-fit g_Na_max and g_K_max were similar from APs with different CPN levels (*n* = 30, 30, and 28 target APs). The voltage dependence and the kinetic profile of the optimal conductance sets are presented in the Extended Data Figure 5-2. ***C***, The native AP waveforms retrieved from APs with different CPN levels were also similar. ***D***, Peak, maximal dV/dt and half-width of the native APs predicted based on recordings with three different CPN settings. Columns show the averages of the native APs, while X denotes the averages of the experimentally measured parameters (*n* = 30, 30, and 28 APs) which were distorted by the instruments.

**Figure 6.**
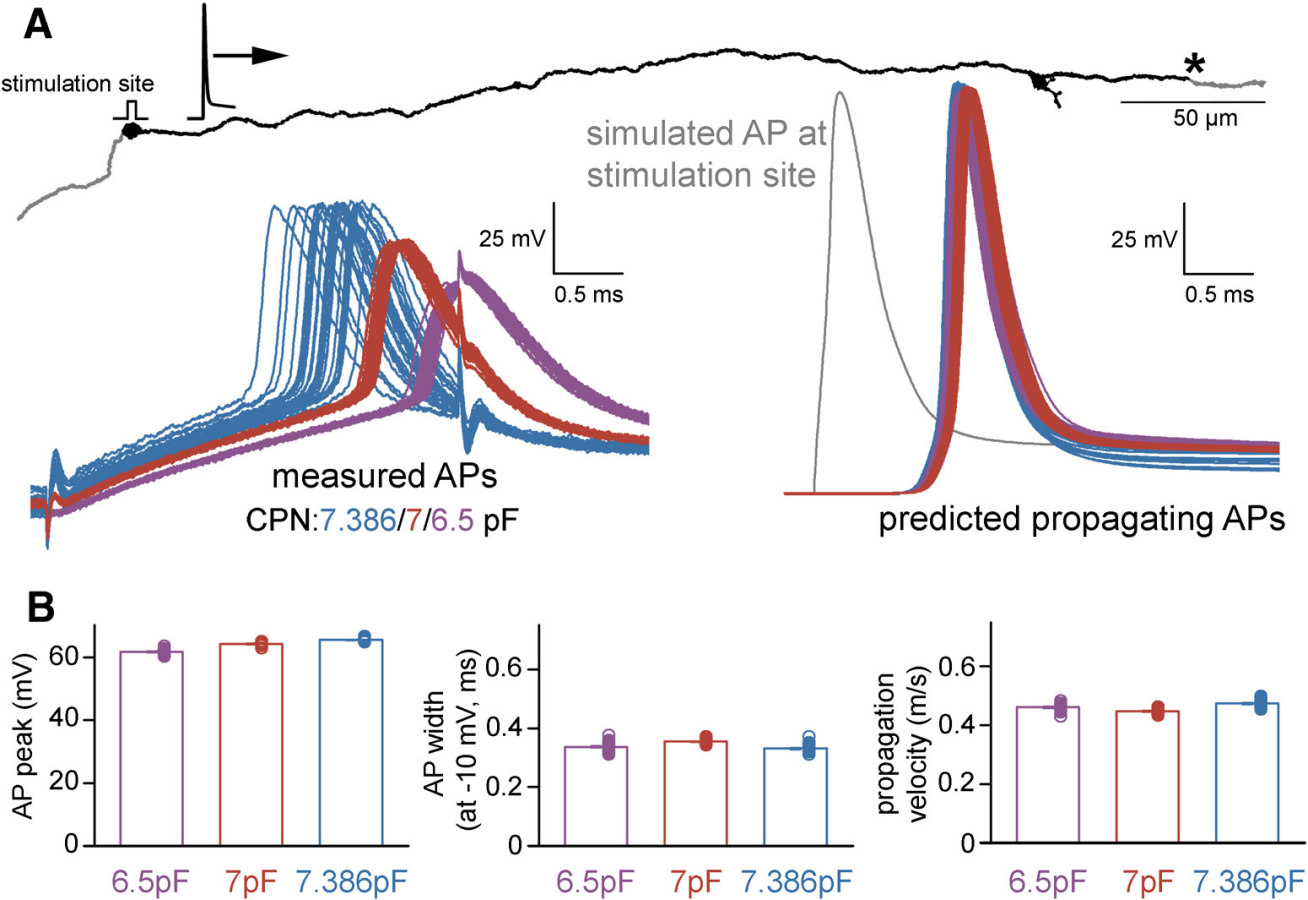
Characteristics of propagating native APs. ***A***, The reconstructed and simulated axonal structure with the position at which native AP parameters were captured (indicated with asterisk) after distal AP initiation (stimulation site) in individual models that were optimized for different recording conditions. Left, 88 original AP recordings that were used for the AP optimization in the models and recorded individually with three different CPN settings (*n* = 30, 30, and 28 with 6.5, 7, and 7.386 pF CPN, respectively). The retrieved conductance sets were applied to the complete axon individually in all 88 cases. The right panel shows the 88 native, propagating simulated APs at the indicated distal point along the axon. The color is as for the measured APs on the left. A simulated AP at the stimulation site is shown in gray. ***B***, Peak, width at −10 mV and propagation velocity of the distally initiated propagating APs retrieved from 88 individual simulations. Note that conventional AP half-width measurements cannot be applied for propagating APs because of the altered apparent threshold.

We used additional experiments to verify the reliability and plausibility of the model predictions. Specifically, we recorded APs with three different CPN settings from the same axon and tested whether it alters the output of the model (CPN = 6.5, 7, and 7.386 pF; Fig. 5*A*). Suboptimal CPN conditions affects the recorded signal in several ways (Extended Data Fig. 5-1). The simulations reproduced the recorded APs with different level of distortions when the CPN in the model was adjusted accordingly (Fig. 5*A*). In addition, the predicted underlying sodium and potassium conductances were also very similar (g_Na_ density: 159.02 ± 1.73, 151.7 ± 0.95, and 160.03 ± 1.47 mS/cm^2^, g_K_ density: 5.83 ± 0.05, 5.41 ± 0.03, and 6.32 ± 0.09 mS/cm^2^; *n* = 30, *n* = 30, and *n* = 28 for CPN = 6.5 pF APs, CPN = 7 pF APs, and CPN = 7.386 pF APs, respectively; Fig. 5*B*; Extended Data Fig. 5-2). Importantly, not only the predicted AP shapes matched but the native APs were also similar despite of the different levels of distortions in the three independent original recording conditions (absolute AP peak: 42.98 ± 0.17, 46.85 ± 0.09, and 47.24 ± 0.14 mV, AP half-width: 0.31 ± 0.003, 0.33 ± 0.002, and 0.34 ± 0.003 ms; *n* = 30, *n* = 30, *n* = 28 for CPN = 6.5 pF APs, CPN = 7 pF APs, and CPN = 7.386 pF APs, respectively; Fig. 5*C*,*D*). For an additional verification, we tested whether the model provides consistent predictions of AP propagation across conductance sets derived from different recording configurations. Specifically, we simulated the natural AP conduction of distally evoked APs to the original recording site (423 μm away; Fig. 6*A*) and measured the speed of propagation and the shape of the incoming APs. The incoming propagating APs were similar (absolute AP peak: 61.73 ± 0.13, 64.22 ± 0.07, and 65.48 ± 0.08 mV, AP width at −10 mV: 0.34 ± 0.003, 0.36 ± 0.002, and 0.33 ± 0.002 ms; *n* = 30, *n* = 30, *n* = 28 for CPN = 6.5 pF APs, CPN = 7 pF APs, and CPN = 7.386 pF APs, respectively; Fig. 6). The prediction of AP propagation velocity is particularly sensitive to model parametrization as the speed of axonal spike propagation strongly depends both on the morphologic properties of the axon and the specific passive and active mechanisms of the axonal membrane. Consistent with previous measurements in hippocampal MFs (Kress et al., 2008; Alle et al., 2009), all model configuration predicted similar propagation speed (0.46 ± 0.002, 0.45 ± 0.001, and 0.47 ± 0.002 m s^−1^; Fig. 6*B*).

10.1523/ENEURO.0059-21.2021.f5-1 Extended Data Figure 5-1 Critical influence of the applied CPN on the recorded AP waveforms. ***A***, Model AP waveforms simulated using the same conductance set but different CPN settings. Notice the AP failure at CPN = 6.2 pF (black) and the oscillation at CPN = 7.6 pF (brown). ***B***, Representative APs recorded (left) and simulated (right) in the same axon with different CPN settings (*n* = 6 APs in each CPN conditions). ***C***, Effects of different CPN level on AP peak (left), threshold (middle) and half-width (right). Gray circles show the AP parameters simulated with the 30 different conductance sets (the same set as in Figure 4). Purple, red and blue symbols show the experimentally recorded AP parameters obtained with three different CPN settings (*n* = 30 APs in each condition). Zero on x-axis represents the originally set 7 pF CPN. Download Figure 5-1, TIF file.

10.1523/ENEURO.0059-21.2021.f5-2 Extended Data Figure 5-2 Gating profile of model conductances obtained from fitting of AP waveforms with different levels of distortions. ***A***, Voltage dependence (left) and kinetic profile (right) of the activation in Na^+^ conductance models obtained by the reconstruction of the APs recorded with different CPN settings (CPN = 6.5 pF in purple, CPN = 7 pF in red and CPN = 7.386 pF in blue). Each line represents single model conductance (*n* = 30/30/28 models). ***B***, Same as in panel ***A*** but for the inactivation of the Na^+^ conductance models. ***C***, Same as in panel ***A*** but for the K^+^ conductance models. Download Figure 5-2, TIF file.

Taken together, these results confirm that complete representation of the recording instruments in a model is sufficient for generating plausible native signals and underlying membrane mechanisms from signals that are distorted by the recording apparatus.

### Instrumental and structural parameters jointly define signal distortion in recordings from small neuronal structures

**Figure 7.**
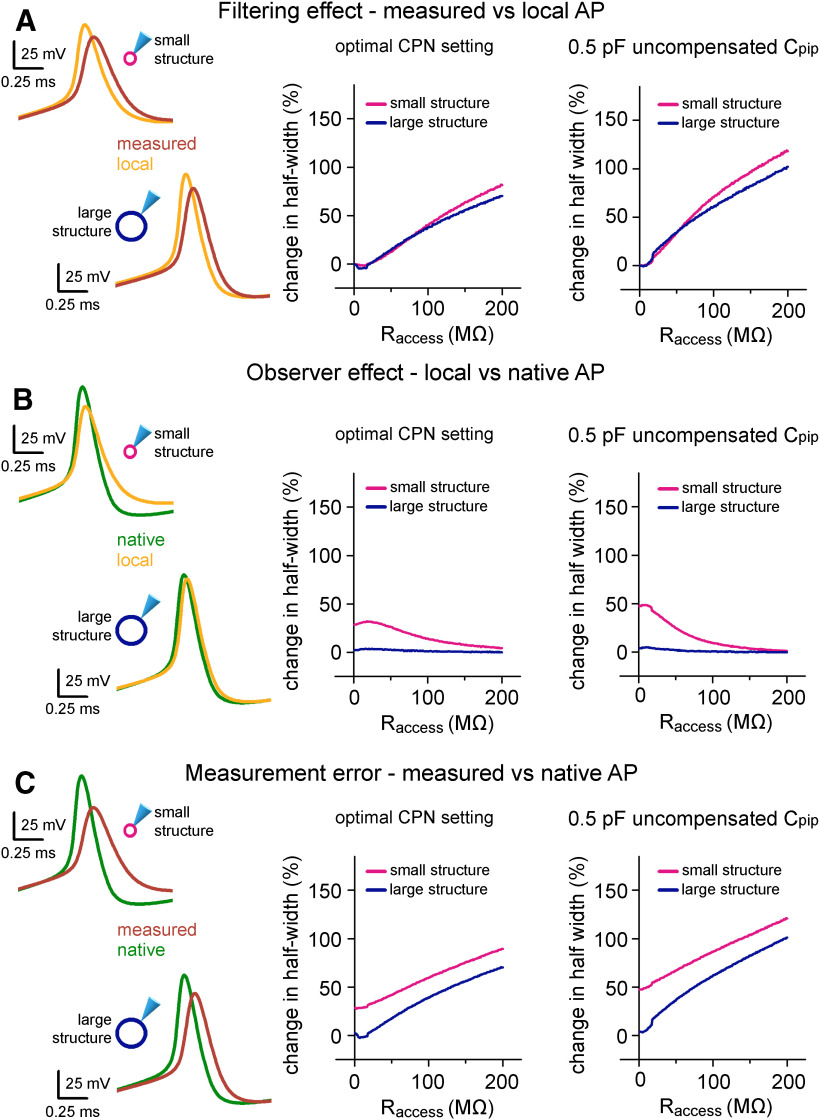
Instrumental and structural parameters cooperatively determine signal distortions in recordings from small neuronal structures. ***A***, On the left, the differences between the waveforms of the same APs within the pipette (measured) and in the two hypothetical cells (local) highlight the filtering effect of 60-MΩ R_access_. Only the size of the membrane surface was different in the two spherical structures, resulting in 1- and 10-pF biological capacitance, which correspond to small axonal and small somatic recordings. Graph in the middle summarizes the filtering effect quantified as the difference in AP half-width over a wide range of R_access_ in the small and large spherical cells. The right graph summarizes the filtering effect in simulations where suboptimal CPN was applied (6.3 pF instead of 6.8 pF). The effect of filtering on the AP amplitude is shown in Extended Data Figure 7-1*A*. ***B***, Using the same simulation environment as in panel ***A***, the observer effect was quantified as the difference between the local AP and the native AP waveform. Thus, these data represent the isolated influence of the instrument on local signal generation. See also Extended Data Figure 7-1*B*. ***C***, To quantify the measurement error, we demonstrate the difference between the measured AP and the native AP waveform in the same conditions as above. Thus, this is the sum of the filtering and observer effects. See also Extended Data Figure 7-1*C*.

In addition to providing a useful tool for predicting and correcting instrumental distortions our simulations confirmed that complete elimination of the instrumental disturbance was not possible during recordings (Ritzau-Jost et al., 2021), since substantial difference persists between the measured and native APs, even when the capacitance compensation reached the maximally attainable level (see Fig. 5*C*). The model raised the possibility that inadequate local signal generation also significantly contribute to the alterations in AP shapes in addition to filtering that affects recordings through patch pipettes. We refer to this effect as observer effect based on the analogy with the concept introduced in the field of physics for situations where the measurement inevitably changes the measured parameter. The observer effect can be quantified as the difference between local APs (signals in the structure when pipette is present) and native APs (signals without the presence of any instrument; Fig. 4*G*,*H*). We quantified the relative contribution of filtering and observer effects to the total instrumental distortion in different experimental situations. Specifically, we varied pipette parameters, compensation settings and the size of the recorded cell (the size of the model neuron was adjusted to match 1- and 10-pF total capacitance, representing an axon or small caliber dendrite and a small cell body, respectively) in a reduced model which included only a single neuronal compartment with the instrumentation and compared the half-widths of APs (Fig. 7).

10.1523/ENEURO.0059-21.2021.f7-1 Extended Data Figure 7-1 Instrumental and structural parameters cooperatively determine signal distortions in recordings from small neuronal structures. The results of the same simulations as in Figure 7, are shown for effects on AP peak. Download Figure 7-1, TIF file.

First, we isolated the filter effect by comparing simultaneous AP signals in the pipette and in the cell (measured vs local AP). In accordance with the general notion, high R_access_ significantly filters fast voltage transients and allows faithful measurement only in a confined R_access_ range (R_access_ that causes 10% AP signal widening: 44 MΩ for small cell and 40 MΩ in case of larger cell; Fig. 7*A*, left). Furthermore, when uncompensated capacitance was added to the circuit by reducing the applied CPN (−0.5 pF), the recording became more vulnerable and reliable recordings needed better R_access_ (R_access_ with 10% distortion: 20 MΩ for small cell and 17 MΩ in case of larger cell; Fig. 7*A*, right).

Next, we isolated the observer effect by comparing the local and native AP shapes with or without the presence of the recording instrument (Fig. 7*B*). The observer effect was always negligible in the case of the larger simulated biological structure as the capacitance added by the pipette was insignificant compared with the cellular parameters. In contrast, when the structure was smaller the structure had to discharge the remaining instrumental capacitance, which was in this case in the range of C_cell_, resulting in significant observer effect on the recorded AP shape (Fig. 7*B*). Consistent with this hypothesis, observer effect was larger in simulations with additional uncompensated C_pip_ (mean change in AP half-width in the R_access_ range of 1–50 MΩ: 29.72 ± 0.23% vs 38.66 ± 1.12%, optimal CPN vs CPN with 0.5 pF uncompensated C_pip_, respectively; Fig. 7*B*). Intriguingly, the observer effect showed a reversed dependence on the R_access_. It was the most pronounced when R_access_ was low. The larger R_access_ presumably isolates the residual instrumental capacitance from the cell and consequently weakens the instrumental impact.

Finally, by comparing the measured and native signals we assessed the overall measurement error. In case of the larger simulated structure, the overall measurement error is dominated by the filtering effect (Fig. 7*C*). In contrast, when C_cell_ is small, the observer effect contributes significantly to the total error. Interestingly, the complementary changes in the two effects make these recordings less sensitive to changes of R_access_ and the error is similar when the R_access_ is low or high (Fig. 7*C*, left). The results confirm that residual uncompensated pipette capacitance further deteriorate the difference between native and measured AP signals (Fig. 7*C*, left). Similar conclusions can be drawn regarding other AP parameters as well (Extended Data Fig. 7-1). Altogether, these model simulations demonstrated that measurement errors in patch-clamp recordings depend not purely on the pipette parameters but the size of the recorded structure itself has influence on the signal distortion.

**Figure 8.**
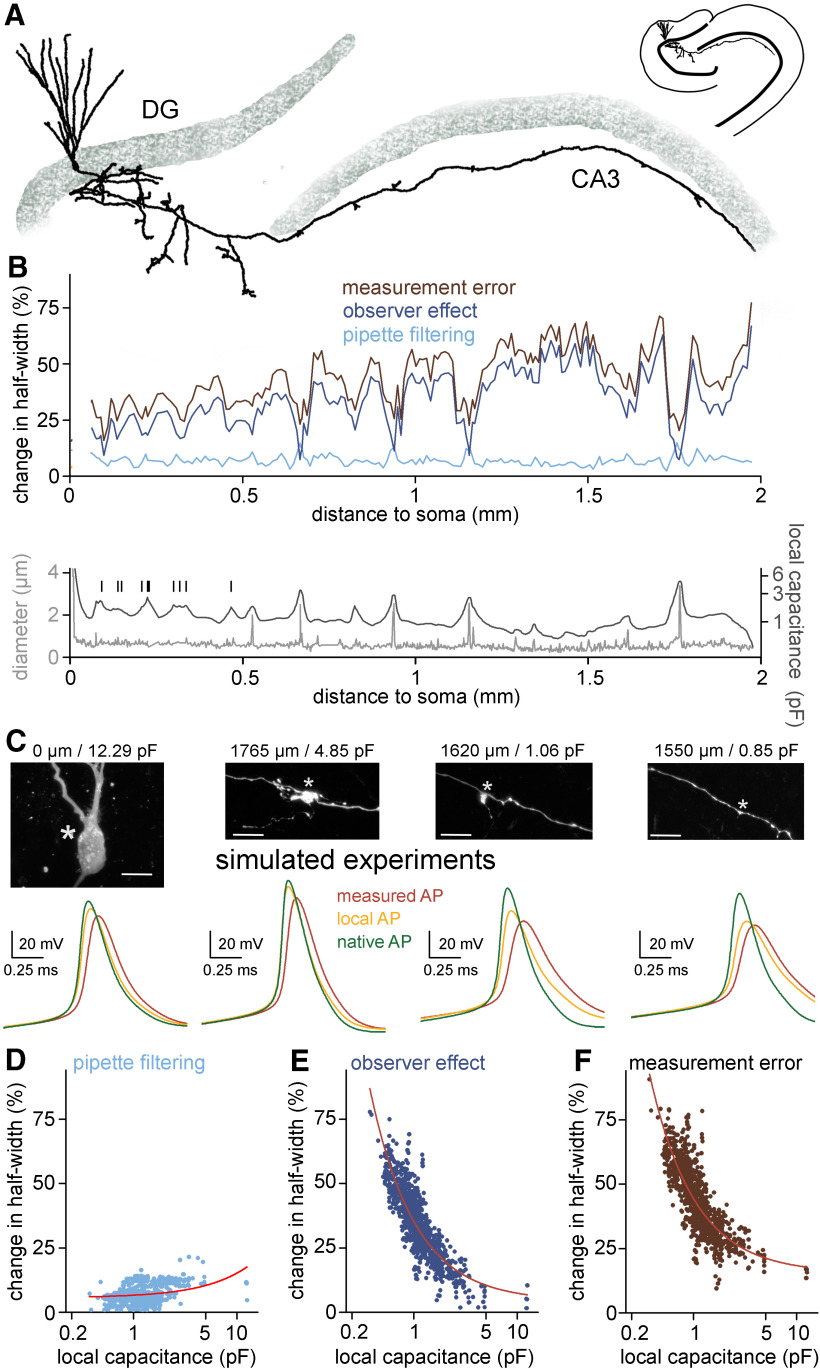
Measurement error along the axon is inhomogeneous and depends on the local biophysical environment. ***A***, Morphology of a somatically labeled complete granule cell that was used for simulating hypothetical recordings along its axon. ***B***, top, Observer and filtering effects (dark blue and light blue, respectively) together with the overall measurement error (brown) in simulated axonal measurements plotted against the somatic distance of the recording position. Each value represent average of five individual measurements in each 10-μm segments. Bottom, Axonal diameter (gray) and local capacitance (black) as a function of somatic distance. Vertical bars indicate the branch points of hilar axon collaterals. Notice that signals that derive from axonal segments that are closer to the soma are less affected by the observer effect because of the capacitive “load” of the somatic membrane. ***C***, Representative simulated recordings from the soma and different axonal sites and confocal images of the simulated recording site (same cell as in panel ***A***). Corresponding somatic distances and local capacitances are indicated on the top. Asterisks mark the recording positions in the simulations. Scale bar: 10 μm. ***D***, The filtering effect showed only weak correlation with the local axonal capacitance (*n* = 941 simulated recordings, *R*^2^ = 0.13, linear fit). Circles represent the filtering effect at independent measurement sites along the same axon. Note the logarithmic scale of the *x*-axis. ***E***, The observer effect showed significant correlation with local axonal capacitance (*n* = 941 simulated recordings, *R*^2^ = 0.75, fitted with power function). ***F***, Correlation between the total measurement error and the local axonal capacitance (*n* = 941 simulated recordings, *R*^2^ = 0.68, fitted with power function).

Finally, to confirm the above findings on the error sources in a native biophysical structure we repeated the above tests in a reconstructed MF that was labeled during somatic recording that ensured more intact axonal arborization (Fig. 8*A*). In this case the simulations ran with fixed instrumental parameters (6.74 pF C_tot_ and 60 MΩ R_access_, with 6.8 pF CPN and 60 MΩ BB correction; Fig. 8*A–C*). We evoked APs along the axon and compared the pipette-measured, local and native AP waveforms to determine the contribution of the observer and filtering effects to the instrumental signal distortion and their dependence on the local biophysical environment (Fig. 8*A–C*). In agreement with the single compartment simulations (Fig. 7), observer effect had a major contribution to the overall measurement error (change in AP half-width: 7.02 ± 1.02%, 34.03 ± 4.59%, and 43.26 ± 4.51% for pipette filtering, for observer effect, and for total measurement error, respectively, *n* = 941 locations; Fig. 8*A–C*). As it is expected from the fixed instrumental parameters, pipette filtering was similar in small and large diameter sections of the axon. In contrast, the size of the observer effect was much larger in smaller axonal structures. Consequently, the measurement error was also the largest in the smallest diameter sections (Fig. 8*B*). Importantly, minima in observer effect coincide with larger local capacitance, which originate from the large mossy fiber boutons in the CA3 area and branching points in the hilus. Indeed, the overall measurement error showed similar inverse correlation to local capacitance (*R*^2^ = 0.68, power) as the observer effect (*R*^2^ = 0.75, power) while the pipette filtering was structure-independent (Fig. 8*D–F*). These findings confirm that measurement error critically depends on the local biophysical environment.

## Discussion

Here, we developed a measurement-based, highly realistic model representation of the recording instrument that faithfully replicates actual patch-clamp recordings when combined with the detailed morphologic reconstruction of the recorded structure. Simulation of the complete experimental condition allows for eliminating the instrumental distortions that are inevitably contaminates signals from small neuronal processes, such as axons. This realistic model also enabled us to determine the extent and sources of perturbations that contaminate electrophysiological signals. The results showed that physical parameters of the measuring instrument and the local biophysical properties of the recorded structure and their quantifiable interactions define the errors in voltage measurements. Consequently, signal deterioration potentially arises from the altered local signal generation instead of pipette filtering in small axonal recordings.

The core hypothesis behind our study is that realistic *in silico* representation of recording instruments together with the detailed morphology and biophysics of the recorded structure provide a better understanding of signal distortions present in direct voltage recordings and offers an applicable offline approach to predict native signals from distorted recordings. The combined simulation of the experimental conditions and the morphology was proved previously to be an ideal tool to describe technical limitations associated with VC measurements and to correct the recorded signals from those distortions (Silver et al., 1992; Spruston et al., 1993; Major et al., 1994; Schaefer et al., 2003; Beaulieu-Laroche and Harnett, 2018). However, these simulations have not included the complete recording instruments, which are known to impose significant distortions on the recorded signals, especially when the biological source is physically small, such as small axons. Other efforts corrected distortions of measured voltage signals by estimating the transfer functions of the specific recording instrument (Magistretti et al., 1998; Brette et al., 2008; Jayant et al., 2017). Although such formulations are computationally efficient, they could not be applied to the variable individual contribution of circuit components (e.g., pipettes, C_pip_ and R_access_ compensatory elements), which imposes different signal distortions, such as filtering or instrumental capacitive load. Here, we implemented the pipette and all amplifier elements as individual circuits as they were actually built in the complete system and showed that the idealized instrument is sufficient to replicate the behavior of the complete measuring instrument and its compensatory capabilities (Figs. 1–3). Thus, we assume that implementing these components will allow adapting the model to other amplifier types.

We characterized the spatial relationship between R_pip_ and C_pip_ along the pipette to create detailed, multicompartmental pipette representation in the model (Fig. 3). Significant pipette resistance can effectively separate downstream pipette capacitance from the amplifier thereby reducing the neutralization capability of the amplifier. For the axonal recording pipettes, we show that the majority of the total C_pip_ originate from the first two and a half mm from the tip. Nevertheless, we found that only a minor fraction of C_pip_ fell into the range where significant R_pip_ was present (Fig. 3*A*). The spatial segregation of R_pip_ and C_pip_ is probably more complete in pipettes with low resistance or with optimized tip geometry. Under such conditions a single RC circuit may be sufficient to describe the electrical behavior of the recording pipettes. We observed decreased relative wall thickness toward the tip that explains the tip-dominated capacitance distribution (Fig. 3*B*). The relative wall thickness was found to be constant previously for borosilicate glass capillaries (Benndorf, 1995). Interestingly, for quartz pipettes, Levis and Rae found similarly decreased R_OI_ at the tip region (Levis and Rae, 1993). On the other hand, optimized pipette fabrication can actually increase the relative wall thickness of quartz capillaries indicating that capacitance distribution critically depends on the pipette material and on the applied pulling process (Dudel et al., 2000).

To outline a feasible application, we used the complete instrumental model to correct the shape of APs directly recorded from a small axon terminal of a hippocampal mossy fiber. The size of this bouton (largest diameter: 0.7 μm) was in the range of typical cortical axon terminals and was much smaller than the famous large mossy fiber terminals. The complete instrumental model in combination with the realistic axonal morphology and biophysics faithfully replicated the measured voltage signals (Figs. 4–6), including the signal artefacts and distorted fast APs. The simulation resulted in ionic currents and conductances that matched with previous results obtained with direct MF recordings from large boutons (Fig. 4*E*,*F*; Geiger and Jonas, 2000; Alle et al., 2009, 2011). Consequently, the retrieved native axonal APs were brief events with large amplitude (Figs. 4-6) whose shape closely resembled the spike waveforms reported for the large axon terminals of the same axon (Geiger and Jonas, 2000; Alle et al., 2009). The simple Hodgkin–Huxley type conductance models with six free parameters were sufficient to restore the experimentally recorded MF AP waveforms. Although APs in the distal MFs can be described with a similar standard Hodgkin–Huxley type gating (Engel and Jonas, 2005; Ohura and Kamiya, 2018), AP simulation in other neuron types or in other subcellular elements required more detailed kinetic schemes (Schmidt-Hieber and Bischofberger, 2010; Hallermann et al., 2012; Ritzau-Jost et al., 2014, 2021). Therefore, implementation of more elaborated conductance models and inclusion of additional conductances can further improve our simulations and adapt to multiple activity regimes, such as the plasticity of AP shapes during sustained activity (Geiger and Jonas, 2000).

The complex model allowed us to dissect the sources of errors that contaminate recordings from small biological structures. As previously described for small, electrotonically compact neurons (D’Angelo et al., 1995; Goodman et al., 1998; Rohrbough and Broadie, 2002), we found that even with careful CPN, the capacitive load added by the recording instrument substantially altered the intrinsic electrical behavior of the axon (Figs. 4, 5). Using a simplified neuronal representation, we confirmed with that instrumental capacitance effectively attenuates the APs in small neuronal structures (Fig. 7) emphasizing again that C_pip_ reduction can substantially improve the accuracy of the measured voltage signal (Sakmann and Neher, 1983; Levis and Rae, 1993; Ogden and Stanfield, 1994; Dudel et al., 2000; Ritzau-Jost et al., 2021). Interestingly, we have shown that increase in the R_access_ reduced the instrumental impact on the cellular electrogenesis (probably because of effective electrical isolation of the neuronal structure from the recording pipette) suggesting that high-impedance recording can have also advantages when experimental subject is small. The high-impedance recordings may reduce the complexity of *post hoc* AP reconstitution as in this case the predicted AP shape depends only on the pipette filtering (Jayant et al., 2017). One of the major findings of our study is that instrumental and cellular factors define the accuracy of CC experiments not in isolation from each other, but their interaction is equally important. This was the most apparent when we examined the cellular contribution of the overall measurement error along a reconstructed MF which forms varicosities with different diameter. Thus, the differently sized axon segments provide different local biophysical conditions (Fig. 8). In this arrangement, which is characteristic to all axons at a various degree (i.e., the size of the terminals and axonal shaft varies), the observed inverse relationship between local capacitance and the measurement error highlights the influence of structural details on direct axonal recordings.

A more general consideration is that the target-size-dependent effects are not specific to the axonal recordings. Experiments that target small cellular structures, whose electrical parameters are comparable to the capacitance introduced by the measuring instrument, are potentially subject to the distortions quantified in our study. The target-dependent measurement errors are not specific to the AP firing either. In fact, the typically high conductance densities in axons (Hu and Jonas, 2014; Ritzau-Jost et al., 2014) can partly compensate for the observer effect. Depending on the local biophysical environment, the recording instruments can induce substantial observer error in dendritic membrane potential as well.

## Synthesis

Reviewing Editor: Alexey Semyanov, Shemyakin-Ovchinnikov Institute of Bioorganic Chemistry Decisions are customarily a result of the Reviewing Editor and the peer reviewers coming together and discussing their recommendations until a consensus is reached. When revisions are invited, a fact-based synthesis statement explaining their decision and outlining what is needed to prepare a revision will be listed below. The following reviewer(s) agreed to reveal their identity: Stefan Hallermann, Maxim Volgushev.

The paper has been now seen by two reviewers, and both have found it of interest. They have agreed that the paper has some important results. The manuscript pays attention to distortion of action potential recordings in small neuronal compartments. Hence correcting the errors by simulation of the pipette and the amplifier have been suggested. Patch-clamp recordings from axonal compartments which the authors have performed are impressive, and the analysis is insightful. Nevertheless, the reviewers have made some comments which warrant minor revision of the manuscript.

Reviewer #1

As explained in the comments to the authors a unique and innovative approach was used that allowed to identify several novel results. In particular, action potential recorded from small axonal structures are highly distorted and simulation of the pipette and the amplifier's circuitry allows correcting for these errors.

The manuscript with the title ‘Small size of recorded neuronal structures confines the accuracy in direct axonal voltage measurements’ describes an impressive and ambitious project. The authors performed direct whole-cell patch clamp recordings from small axonal compartments and thoroughly analysed the errors of current clamp recordings. Particularly, the authors dissected the filtering of the voltage signal by the pipette and the amplifier (termed filtering effect) and the distortion of the voltage signal itself by the measuring apparatus (termed observer effect). The insightful analysis was possible by an innovative and, to my knowledge, unique approach of modelling the pipette and entire amplifier circuitry within the simulation environment NEURON. This is an heroic and elaborative approach. To this end, the authors even performed longitudinal grinding of their glass pipettes. The approach was validated by various attempts including intentional misadjustment of the pipette capacitance circuitry of the amplifier resulting in astonishingly similar reconstructed native action potentials. The most exciting take home messages are the large and rapid action potentials, the profound distortions by the current clamp measurements, and the dissection of the above-mentioned filtering and observer effect. I fully support publication and have only the following tiny suggestions.

The formatting of the references is sometimes inconsistent in terms of abbreviations with ‘et al.’ or in terms of providing the first as well as family name of the first author in some cases.

Fig. 4-1 E:

Maybe ‘vs. native AP st8’ could be removed in the labelling as the color coding is inconsistent with the color code of axes labelling.

Line 64:

Please add the name of the university.

Line 326-329: The sentence is grammatically wrong and/or difficult to understand. Consider re-wording.

Line 398: The variable capacitor was added to the back of the pipette. The exceptional quality of the fits justifies this approach. Maybe the authors could speculate why this approach works despite the fact that the tip of the pipette seems to dominate the capacitance of the pipette (Fig. 3A). I guess I am wondering if the distributed multicompartment pipette representation is required for the approach.

Line 437:

Are the provided values for the maximum rate of rise correct? They appear very different compared with the other parameters.

Line 604-606: In this context, it could be noted that heating periods without pulling during the pipette fabrication process can in contrast increase the relative wall thickness as described in the cited paper Dudel et al. (2000).

Reviewer #2

An interesting paper addressing a very important and actual technical problem - how recording apparatus disturbs recording, and most importantly - also generation of electrical signals (specifically, APs) in small structures. The authors build a model that includes components of recording apparatus (amplifier components, electrodes, etc.) that introduce these distortions, fit the model to measured parameters and then to recorded APs. Removal of the recording apparatus from the model allows then to assess the 'native' AP, i.e. how it would be if recording electrode were not present.

The study is carefully carried out and the paper is well written.

However, there are some points that needed to be clarified. Reasons for choosing model parameters is often not clear; especially in cases when basic parameters differ by a factor of 10x or 100x in different simulations.

There are also some comments and suggestions on stile.

(throughout the text): citations - please unify, no names/initials

Comments below are 'by line'; they are not sorted by importance.

l. 7/8 ... will precisely reveal signal distortions and provides a tool that predicts...

-> will reveal ... and provide ...

l.22 subsequent removal from distorted recordings

(may be) -> subsequent reconstruction of a 'native' signal that would be generated in that structure without the distortion by recording

(or, defining 'native' somewhere else in the Abstract or Significance Statement)

l. 42 ... The shape of axonal APs is a key

43 determinant of neuronal signaling that affects neurotransmitter release and short-term dynamics in synaptic

44 connections ...

Yes, but next step, translation of MP changes into spike trains is as important for encoding in neuronal networks as synaptic transmission. Both theoretical studies and experimental evidence demonstrated the dependence of encoding on AP generation/shape. Please add.

l.56 ... with the aim

57 of subsequent, simulated removal of the instrumental contributions and distortions from the recorded signals.

-> (may be?) with the aim of subsequent recovery of undistorted ('native') signals by removing instrumental contributions from the model

l. 100 Bessel filters. ... to match of our actual biological -> to match our actual biological

l. 155 ... Patch pipette... - move to l. 145, before pipette solution?

l. 184 The apparent resting membrane potential of the recorded MF axon... not clear, what 'apparent' means here

l. 218 ... exploration substantial part -> exploration of substantial part

l. 249 ... Hieber & Bischofberger, 2010) in a single compartment neuron...

please provide parameters of the model;

Also, more generally, a table with all parameters of all models used would be helpful.

l. 258 The axonal diameters were either homogeneously increased by 160 nm or reduced by 180 nm...

This is somewhat counterintuitive procedure for modelling shrinking; I may be wrong, but proportional shrinking feels more plausible.

l. 273 Electrical behavior of small and larger cells was set by scaling the active and passive biophysical parameters of

274 the compartment (for small cell: Cm=10 μF/cm2, Rm=2 kΩ/cm2, gNa density=1.5 S/cm2, gK density=0.4 S/cm2; for

275 larger cell: Cm=100 μF/cm2, Rm=0.2 kΩ/cm2, gNa density=15 S/cm2, gK density=4 S/cm2). To keep Rseal/Rcell ratio

276 constant between the two conditions, Rseal was set to 50 GΩ and 5 GΩ, for small and larger cell, respectively.

The choice of parameters above is not clear.

Please explain

(i) why are they 10x - 100x fold different from those estimated e.g. l427-428; or implemented in cell model l.282-283;

(ii) why channel density is 10x higher in a larger structure?

Also 10x higher Rseal in small structures is counterintuitive: it might be related to quality of initial gigaseal during patching, which might be better (higher Rseal) for large structures that are easier to patch. Please explain.

l. 345 ... -> Amplifiers

l. 458 statistics, p = 6.88*10**(-60), - I am not sure that for p<<0.001 (or p<<0.0001) exact value of p has any meaning; (same for other comparisons with p<<0.0001)

Also, even if to take difference between 10**(-29) and 10**(-60) seriously, statement that

l. 461 .... the upstroke of spikes was the most different between local

462 and measured APs as it is the most sensitive to...

is formally wrong: 10**(-29) > 10**(-60)

Apart from that, I thought ANY filtering could only decrease the amplitude, increase width, etc; and paired comparison will always be highly significant. It feels like comparing (x) to (0.9x).

l. 466 Our model predicts that APs of the small MF bouton had lower peak and slower kinetics than APs measured from

467 large MF terminals (Henrik Alle et al., 2009; Geiger & Jonas, 2000)....

This reads as if 'your model' is in the cited papers;

Also, your model had not yet predicted this by line 466; it will consider this topic later (Fig 7 and related text)

l. 472 ... Thus, we predict how the native APs would look if the recording instrument was not present.

Please consider to define 'native' early in the paper; 'native' is already used in the text several times.

l. 476 30 APs) compared to the local spikes modeled with the experimental rig. The obtained native AP parameters were

477 similar to those reported from large MF boutons,...

Were native APs from small MFs were compared to recorded APs from large MFs?

this does not feel right... it implies that recordings from large MFs were not distorted/filtered at all;

l. 524 ... the size of the recorded cell (size was set to as Ccell = 1 pF and 10 pF, corresponding

525 to the size of axons or small caliber dendrites and small cell bodies, respectively)

'size' in pF is jargon, please correct.

l. 529 .. significantly filters fast

530 ... and allowed ... -> allows

l. 603 ... The gradual increase

604 of relative wall thickness from the tip explains the larger contribution of the tip.

while technically correct, this sounds strange, what about:

-> The gradual decrease of relative wall thickness toward the tip explains the larger contribution of the tip....

l. 615 ... resembled to the spike waveforms -> resembled the spike waveforms

l. 640 -> segments provide

l. 643 and the measurement error highlight the importance of structural details on direct axonal recordings.

... importance ... of...

or, ... importance of the influence ... on...

l. 844 . -> Asterisk marks

Table 1, passive properties and conductance densities for thin/thick morphology.

Were parameters adjusted to keep (what? currents? Rinput? else?) constant?

If electrical characteristics of a model cell are kept independent of cell size:

(i) what is the point of changing cell size?

(ii) the fact that APs remain about same is not really surprising?

I would understand if this would be done for estimating possible range of (R, C, g...) per cm2, to account for possible deformations of tissue during morphological processing, but this does not seem to be the point.

Or am I missing something here?

## Author Response

Dear Dr. Semyanov,

Please find here the revised version of our manuscript entitled ‘Small size of recorded neuronal structures confines the accuracy in direct axonal voltage measurements". We thank the reviewers for their supportive words and constructive suggestions. We followed the helpful advices of the reviewers and changed the manuscript accordingly. We hope that the revised manuscript is now suitable for publication in eNeuro.

Below are our point-by-point responses to their comments (responses are in italic).

Yours sincerely,

János Brunner

Reviewer #1

As explained in the comments to the authors a unique and innovative approach was used that allowed to identify several novel results. In particular, action potential recorded from small axonal structures are highly distorted and simulation of the pipette and the amplifier's circuitry allows correcting for these errors.

The manuscript with the title ‘Small size of recorded neuronal structures confines the accuracy in direct axonal voltage measurements’ describes an impressive and ambitious project. The authors performed direct whole-cell patch clamp recordings from small axonal compartments and thoroughly analysed the errors of current clamp recordings. Particularly, the authors dissected the filtering of the voltage signal by the pipette and the amplifier (termed filtering effect) and the distortion of the voltage signal itself by the measuring apparatus (termed observer effect). The insightful analysis was possible by an innovative and, to my knowledge, unique approach of modelling the pipette and entire amplifier circuitry within the simulation environment NEURON. This is an heroic and elaborative approach. To this end, the authors even performed longitudinal grinding of their glass pipettes. The approach was validated by various attempts including intentional misadjustment of the pipette capacitance circuitry of the amplifier resulting in astonishingly similar reconstructed native action potentials. The most exciting take home messages are the large and rapid action potentials, the profound distortions by the current clamp measurements, and the dissection of the above-mentioned filtering and observer effect. I fully support publication and have only the following tiny suggestions.

We appreciate the reviewer's positive comments and helpful suggestions.

1)

The formatting of the references is sometimes inconsistent in terms of abbreviations with ‘et al.’ or in terms of providing the first as well as family name of the first author in some cases.

Done.

2)

Fig. 4-1 E:

Maybe ‘vs. native AP st8’ could be removed in the labelling as the color coding is inconsistent with the color code of axes labelling.

We modified the labelling on the Fig.4-1 E as suggested.

3)

Line 64:

Please add the name of the university.

Done.

4)

Line 326-329: The sentence is grammatically wrong and/or difficult to understand. Consider re-wording.

We reworded the corresponding part of the manuscript that now reads as follows:

’ We used idealized circuits for model amplifier construction to start with, namely, a resistive feedback circuit (current-to-voltage converter) for the VC and a voltage follower circuit (a unity gain voltage buffer) with idealized current source for the CC."

5)

Line 398: The variable capacitor was added to the back of the pipette. The exceptional quality of the fits justifies this approach. Maybe the authors could speculate why this approach works despite the fact that the tip of the pipette seems to dominate the capacitance of the pipette (Fig. 3A). I guess I am wondering if the distributed multicompartment pipette representation is required for the approach.

We thank the reviewer for raising this point. We found that majority of the pipette capacitance originates from the first 2-3 mm of the pipette. However, the resistance of the pipette was even more confined to the tip region (Fig 3A) leading to effective spatial segregation of Cpip and Rpip. We fitted the measured Rpip data with an exponential function to quantify the spatial distribution of the pipette resistance. This fit revealed that the pipette resistance drops with a length constant of 208 um and falls below 1 MOhm after 703 um. We also found that only a small fraction of total Cpip originated from regions where significant Rpip was present (15.3% at 700 um tip distance, 0.48{plus minus}0.02 pF from the total of 3.1{plus minus}0.04 pF) (Fig. 3A). These results show that the majority of the total Cpip was practically separated from the pipette resistance in these small recording pipettes. The high degree of spatial segregation of Rpip and Cpip allowed us to simplify the Cpip adjustment in the model by using the variable capacitor at the back of the pipette. Notably, the separation of Rpip and Cpip is probably more prominent in conventional somatic patch pipettes because of their smaller resistance and favorable morphology (the resistance presumably drops more sharply in such pipettes as a result of the larger cone angle in the tapered region). In such conditions, a single RC circuit may be sufficient to describe pipettes.

We revised the text to emphasize the spatial separation of Rpip and Cpip and to include the rationale of the position of Cpip adjustment both in the Results section as well as in the Discussion:

In the Results section:

‘Rpip declines from the tip with length constant of 208 {plus minus} 40 μm and fall below 1 MΩ at 703 μm tip distance, exponential fit of the data shown on Fig. 3A, R2=0.86, n = 55 resistance measurements;..."

‘...suggesting inhomogeneous capacitance distribution along pipettes (0.03 {plus minus} 0.01 pF, 0.48 {plus minus} 0.02 pF, 2.52 {plus minus} 0.05 pF and 3.1 {plus minus} 0.04 pF at 0.1 mm, 0.7 mm, 3 mm and 7 mm tip distances respectively, n=39, 39, 40 and 38 measurements, Figure 3A)."

‘ Model pipette parameters (Rpip and Cpip) can be adjusted using an additional resistor and capacitor to account for variability across individual pipettes. Since the bulk of pipette capacitance is spatially separated from the pipette resistance and because these pipettes has similar shape, we placed the tunable resistor to the tip and the capacitor to the back of the pipette model (Rtip and Cbase on Figure 3C)."

In the Discussion:

‘We characterized the spatial relationship between Rpip and Cpip along the pipette to create detailed, multicompartmental pipette representation in the model (Figure 3). Significant pipette resistance can effectively separate downstream pipette capacitance from the amplifier thereby reducing the neutralization capability of the amplifier. For the axonal recording pipettes, we show that the majority of the total Cpip originate from the first two and a half mm from the tip. Nevertheless, we found that only a minor fraction of Cpip fell into the range where significant Rpip was present (Fig. 3A). The spatial segregation of Rpip and Cpip is probably more complete in pipettes with low resistance or with optimized tip geometry. Under such conditions a single RC circuit may be sufficient to describe the electrical behavior of the recording pipettes."

6)

Line 437:

Are the provided values for the maximum rate of rise correct? They appear very different compared with the other parameters.

We thank the reviewer for pointing out this error. We corrected this value in the text (see below) and also re-checked other data values within the manuscript for potential errors of similar origin.

"...maximum rate of rise: 363.26 {plus minus} 0.93 V/s vs. 355.23 {plus minus} 9.39 V/s, simulated vs. recorded APs, respectively... ‘

7)

Line 604-606: In this context, it could be noted that heating periods without pulling during the pipette fabrication process can in contrast increase the relative wall thickness as described in the cited paper Dudel et al. (2000).

We now mention this point in the Discussion:

‘We observed decreased relative wall thickness toward the tip that explains the tip-dominated capacitance distribution (Fig. 3B). The relative wall thickness was found to be constant previously for borosilicate glass capillaries (Benndorf 1995). Interestingly, for quartz pipettes, Levis and Rae found similarly decreased ROI at the tip region (Levis and Rae 1993). On the other hand, optimized pipette fabrication can actually increase the relative wall thickness of quartz capillaries indicating that capacitance distribution critically depends on the pipette material and on the applied pulling process (Dudel, Hallermann et al. 2000).’

Reviewer #2

An interesting paper addressing a very important and actual technical problem - how recording apparatus disturbs recording, and most importantly - also generation of electrical signals (specifically, APs) in small structures. The authors build a model that includes components of recording apparatus (amplifier components, electrodes, etc.) that introduce these distortions, fit the model to measured parameters and then to recorded APs. Removal of the recording apparatus from the model allows then to assess the 'native' AP, i.e. how it would be if recording electrode were not present.

The study is carefully carried out and the paper is well written.

However, there are some points that needed to be clarified. Reasons for choosing model parameters is often not clear; especially in cases when basic parameters differ by a factor of 10x or 100x in different simulations.

There are also some comments and suggestions on stile.

(throughout the text): citations - please unify, no names/initials

Comments below are 'by line'; they are not sorted by importance.

We thank the reviewer for the valuable comments and constructive suggestions.

1)

(throughout the text): citations - please unify, no names/initial

Done.

2)

l. 7/8 ... will precisely reveal signal distortions and provides a tool that predicts...

-> will reveal ... and provide ...

Done.

3)

l.22 subsequent removal from distorted recordings

(may be) -> subsequent reconstruction of a 'native' signal that would be generated in that structure without the distortion by recording

(or, defining 'native' somewhere else in the Abstract or Significance Statement)

We have defined the term 'native' in the Abstract and in the Significance statement as well.

In the abstract:

‘... provide a tool that predicts native, instrument-free electrical signals from distorted voltage recordings.’

In the Significance Statement:

"... allowing not only the quantification of errors but also the reconstruction of native signals that would be generated in the excitable structure without instrumental distortion. ‘

4)

l. 42 ... The shape of axonal APs is a key

43 determinant of neuronal signaling that affects neurotransmitter release and short-term dynamics in synaptic

44 connections ...

Yes, but next step, translation of MP changes into spike trains is as important for encoding in neuronal networks as synaptic transmission. Both theoretical studies and experimental evidence demonstrated the dependence of encoding on AP generation/shape. Please add.

We thank to reviewer for raising this point. We added the following text to the Introduction:

"This synaptic activity generates dynamic changes in the postsynaptic membrane potential that would be translated into action potential firing of the postsynaptic neuron (Koch and Segev 2000, London and Häusser 2005, Silver 2010). Therefore, accurate measurement of the membrane voltage is essential..."

5)

l.56 ... with the aim

57 of subsequent, simulated removal of the instrumental contributions and distortions from the recorded signals.

-> (may be?) with the aim of subsequent recovery of undistorted ('native') signals by removing instrumental contributions from the model

Thank you for the suggestion, which is now included as follows:

Using this complex model we simulated actual recording conditions with the aim of subsequent recovery of undistorted (native) signals by removing instrumental contributions from the model.

6)

l. 100 Bessel filters. ... to match of our actual biological -> to match our actual biological

Done.

7)

l. 155 ... Patch pipette... - move to l. 145, before pipette solution?

Done.

8)

l. 184 The apparent resting membrane potential of the recorded MF axon... not clear, what 'apparent' means here

We used the term ‘apparent resting membrane potential’ to emphasize that the measured resting membrane potential is contaminated by the depolarizing effect produced by the leak current flowing through the seal.

We have removed the term ‘apparent’ to avoid potential confusions:

’ The measured resting membrane potential of the recorded MF axon was -75.2 mV....

9)

l. 218 ... exploration substantial part -> exploration of substantial part

Done.

10)

l. 249 ... Hieber & Bischofberger, 2010) in a single compartment neuron...

please provide parameters of the model;

Done.

‘...we generated AP waveforms using independent, sophisticated sodium and potassium conductance mechanisms using 8-state kinetic schemes obtained from (Schmidt-Hieber and Bischofberger 2010, Hallermann, De Kock et al. 2012) in a single compartment neuron (Cm: 1 μF/cm2, Rm: 10 kΩ/cm2, Ri: 150 Ω*cm, diameter of the compartment:15 um). Channel densities and kinetics were systematically varied (gmaxNa8st range: 150-450 mS/cm2, gmaxK8st range: 250-850 mS/cm2, channel kinetics were scaled by a factor of 2-4) to generate diverse AP shapes (AP peak range: 19.28-65.82 mV, AP half-width range: 0.21-0.62 ms). We next simulated CC measurements with these conductance sets to generate noisy targets (Raccess= 100 MΩ, Cpip=6.74 pF, BB compensation=100 MΩ, CPN compensation= 6.8 pF, Rseal=50 GΩ, added noise SD: 0.45 mV) for subsequent optimization. Our standard fitting routine reliably retrieved the native AP shapes."

11)

Also, more generally, a table with all parameters of all models used would be helpful.

The revised manuscript contains a summary table of the model parameters used (as Table 1).

12)

l. 258 The axonal diameters were either homogeneously increased by 160 nm or reduced by 180 nm...

This is somewhat counterintuitive procedure for modelling shrinking; I may be wrong, but proportional shrinking feels more plausible.

We reasoned that two possible sources of error could cause distortions in our anatomical reconstruction: (1) tissue shrinkage caused by post-hoc histological processing, (2) overestimation of diameters due to the resolution limit of light microscopy. With the homogeneous manipulation of the axonal diameter we primarily aimed to explore potential errors caused by the uncertainty in the diameter estimations rather than errors associated with the shrinkage. We agree that proportional increase of the axonal diameters is a more appropriate way to explore shrinkage-related issues than scaling with absolute values. Therefore, we have performed additional control simulations in which the axonal diameters were proportionally changed by 30%. Similar to the homogenous diameter manipulation these new control simulations support the major findings of the manuscript (this data are included in Table 2).

The updated text now reads as follows:

‘To evaluate the potential effects of inaccurate anatomical representation of the recorded axon caused by tissue shrinkage or imperfect optical resolution, we artificially altered the reconstructed morphologies and tested how the recovered native APs were affected. For testing the impact of inaccurate diameter estimation, axonal diameters were either homogeneously increased by 160 nm or reduced by 180 nm (axons were not allowed to shrink below 100 nm). In another control simulation we proportionally increased the axonal diameters by 30% to compensate for potential tissue shrinkage.’

13)

l. 273 Electrical behavior of small and larger cells was set by scaling the active and passive biophysical parameters of

274 the compartment (for small cell: Cm=10 μF/cm2, Rm=2 kΩ/cm2, gNa density=1.5 S/cm2, gK density=0.4 S/cm2; for

275 larger cell: Cm=100 μF/cm2, Rm=0.2 kΩ/cm2, gNa density=15 S/cm2, gK density=4 S/cm2). To keep Rseal/Rcell ratio

276 constant between the two conditions, Rseal was set to 50 GΩ and 5 GΩ, for small and larger cell, respectively.

The choice of parameters above is not clear.

Please explain

(i) why are they 10x - 100x fold different from those estimated e.g. l427-428; or implemented in cell model l.282-283;

(ii) why channel density is 10x higher in a larger structure?

We would like to emphasize that unlike the models that had realistic axonal morphology (models described in l.427-428 and l.282-283 of the previous version of the manuscript), the simulations related to the section ‘Instrumental and structural parameters jointly define signal distortion in recordings from small neuronal structures ‘ (this is the model described in l. 273-276) were performed on a single-compartment, isopotential neuron representation. We have chosen this simplified representation for these simulations because we could quantify the size of the modelled neuron with a single value (with its total capacitance).

Note that due to its isopotential behaviour, single compartment neuron representation ignores the spatial dimension as the total capacitance and the sum of total ionic currents define the membrane voltage homogeneously, in each point of the membrane. There are two different potential approaches to set total capacitance and total ionic currents in a perfectly isopotential neuron. We can adjust the size of the compartment, while specific channel densities are kept constant. Alternatively, we can keep the size of the compartment fixed and adjust the specific density values of the implemented mechanisms. As the primary goal of this particular experiment was to determine the dependence of mechanisms on the biophysical size of structure in a well-controlled environment, this latter approach was chosen in the previous version of the manuscript to create a small neuron with total capacitance of 1 pF and a larger neuron with total capacitance of 10 pF (see below for the alternative solution). We fixed the size of the compartment to 10 μm2 surface area and set the axial resistance to a nominally zero value (1e-9 Ω*cm). For the small cell, we set the specific membrane mechanisms to:

Cm=10 μF/cm2, Rm=2 kΩ/cm2, gNa density=1.5 S/cm2, gK density=0.4 S/cm2

Note that this representation is equivalent to an isopotential cell with 100 μm2 surface area and specific mechanisms of: Cm=1 μF/cm2, Rm=20 kΩ/cm2, gNa density=150 mS/cm2, gK density=40 mS/cm2.

To simulate a larger cell, we scaled specific membrane mechanisms by a factor of 10:

Cm=100 μF/cm2, Rm=0.2 kΩ/cm2, gNa density=15 S/cm2, gK density=4 S/cm2.

Again, this representation is equivalent to an isopotential cell with 1000 μm2 surface area and specific mechanisms of Cm=1 μF/cm2, Rm=20 kΩ/cm2, gNa density=150 mS/cm2, gK density=40 mS/cm2.

Altogether, we argue that the biophysical properties of the single-compartment neuron representations described in l. 273-276 were consistent with those of the morphologically detailed neurons presented throughout the manuscript.

Nevertheless, to unify model parametrizations throughout the manuscript, we repeated simulations related to the section ‘Instrumental and structural parameters jointly define signal distortion in recordings from small neuronal structures’ with fixed specific membrane parameters. In this case, specific membrane mechanisms were fixed to: Cm=1 μF/cm2, Rm=20 kΩ/cm2, gNa density=150 mS/cm2, gK density=40 mS/cm2 and only the surface area of the neuron differed between the small and the larger cell (100 μm2 and 1000 μm2, to set 1 pF and 10 pF total capacitance, respectively). The data in the Results, on Figure 7 and on Figure 7-1 were updated according to these new simulations.

The model description in the Materials and Methods section now reads as follows:

"We used a single-compartment neuron in these simulations (Cm: 1 μF/cm2, Rm: 20 kΩ/cm2, gmaxNa: 150 mS/cm2, gmaxK: 40 mS/cm2). Surface area of the compartment was set to 100 μm2 or 1000 μm2, to model a small neuronal structure with 1 pF total capacitance or a larger cell with 10 pF total capacitance, respectively.’

Also 10x higher Rseal in small structures is counterintuitive: it might be related to quality of initial gigaseal during patching, which might be better (higher Rseal) for large structures that are easier to patch. Please explain.

With identical Rseal, the effect of seal on the electrical behavior of the recorded neuron would be different depending on the size of the structure. For example, the resting membrane potential of the small structure would be more depolarized compared to those of the larger structure (see Perkins 2006, Wilson et al. 2011). Although this seal-induced voltage shift can be compensated by constant current injection, the shapes of action potentials would be also different as the contribution of the current flowing through the seal to the total ionic current would be larger in the small neuron.

For these initial simplified modelling we decided to keep constant the ‘impact’ of the seal rather than its absolute value between the models. Therefore, Rseal was reduced tenfold (to 5GOhm) in the simulations with larger structure. With this adjustment the Rseal/Rcell ratio, as well as the contribution of the seal current to the total ionic current, was identical irrespective of the size of the modeled structure. The sentence has been rewritten to clarify the reason of the different Rseal:

’ To keep the seal-induced signal distortion constant in the two conditions, Rseal was reduced to 5 GΩ, for the large cell, which results in similar Rseal/Rcell ratio as for the small structure."

Nevertheless, empirical observations also support the applied difference in Rseal as smaller tip size of the pipettes typically used in recordings from small structures is favorable for the high quality seal formation (Malboubi,& Jiang, 2014).

14)

l. 345 ... -> Amplifiers

Done.

15)

l. 458 statistics, p = 6.88*10**(-60), - I am not sure that for p<<0.001 (or p<<0.0001) exact value of p has any meaning; (same for other comparisons with p<<0.0001)

Also, even if to take difference between 10**(-29) and 10**(-60) seriously, statement that

l. 461 .... the upstroke of spikes was the most different between local

462 and measured APs as it is the most sensitive to...

is formally wrong: 10**(-29) > 10**(-60)

Apart from that, I thought ANY filtering could only decrease the amplitude, increase width, etc; and paired comparison will always be highly significant. It feels like comparing (x) to (0.9x).

We accept the reviewer's point that statistical significance will be inherently high in pairwise comparisons of filtered vs. unfiltered parameters. We have removed filtering-related statistical descriptions from the text.

For the comparison of native vs local AP parameters we now report the statistical significances with p<<0.0001 instead of the exact values.

16)

l. 466 Our model predicts that APs of the small MF bouton had lower peak and slower kinetics than APs measured from

467 large MF terminals (Henrik Alle et al., 2009; Geiger & Jonas, 2000)....

This reads as if 'your model' is in the cited papers;

Also, your model had not yet predicted this by line 466; it will consider this topic later (Fig 7 and related text)

We removed this sentence. Please see the response to comment #18 for justification.

17)

l. 472 ... Thus, we predict how the native APs would look if the recording instrument was not present.

Please consider to define 'native' early in the paper; 'native' is already used in the text several times.

The term 'native' has been defined early in the manuscript (Please see the response to comment #3).

18)

l. 476 30 APs) compared to the local spikes modeled with the experimental rig. The obtained native AP parameters were

477 similar to those reported from large MF boutons,...

Were native APs from small MFs were compared to recorded APs from large MFs?

this does not feel right... it implies that recordings from large MFs were not distorted/filtered at all;

Here we intended to compare the reconstructed native APs from small boutons with the previously recorded APs from large boutons. We agree with the reviewer's opinion concerning the drawbacks of the direct comparison of the predicted small MF APs with recorded large MF APs.

We have removed this comparison from the manuscript.

19)

l. 524 ... the size of the recorded cell (size was set to as Ccell = 1 pF and 10 pF, corresponding

525 to the size of axons or small caliber dendrites and small cell bodies, respectively)

'size' in pF is jargon, please correct.

Done.

’ ... (the size of the model neuron was adjusted to match 1 pF and 10 pF total capacitance, representing an axon or small caliber dendrite and a small cell body, respectively)..."

20)

l. 529 .. significantly filters fast

530 ... and allowed ... -> allows

Done.

21)

l. 603 ... The gradual increase

604 of relative wall thickness from the tip explains the larger contribution of the tip.

while technically correct, this sounds strange, what about:

-> The gradual decrease of relative wall thickness toward the tip explains the larger contribution of the tip....

The text has changed. Please see the response to comment #5 of reviewer 1.

"... For the axonal recording pipettes, we show that the majority of the total Cpip originate from the first two and a half mm from the tip. ... ‘

’ ...We observed decreased relative wall thickness toward the tip that explains the tip-dominated capacitance distribution (Fig. 3B)."

22)

l. 615 ... resembled to the spike waveforms -> resembled the spike waveforms

Done.

23)

l. 640 -> segments provide

Done.

24)

l. 643 and the measurement error highlight the importance of structural details on direct axonal recordings.

... importance ... of...

or, ... importance of the influence ... on...

Done.

"the observed inverse relationship between local capacitance and the measurement error highlights the influence of structural details on direct axonal recordings"

25)

l. 844 . -> Asterisk marks

Done.

26)

Table 1, passive properties and conductance densities for thin/thick morphology.

Were parameters adjusted to keep (what? currents? Rinput? else?) constant?

If electrical characteristics of a model cell are kept independent of cell size:

(i) what is the point of changing cell size?

(ii) the fact that APs remain about same is not really surprising?

I would understand if this would be done for estimating possible range of (R, C, g...) per cm2, to account for possible deformations of tissue during morphological processing, but this does not seem to be the point.

Or am I missing something here?

The data shown in this table (it is Table 2 now) summarizes the results of the control simulations, in which we addressed the potential systematic errors in model predictions.

(i)With the thin/thick morphology controls, we assessed possible effects of incorrect morphological representation (primarily due to the erroneous diameter estimations, please see also the response #11).

(ii) Using these altered morphologies, we independently repeated the consecutive optimization steps to retrieve cellular parameters necessary for the AP reconstitution.

For each morphology, we first predicted the passive membrane parameters and then the active conductances by fitting the model responses to the experimentally recorded data. Hence, the recovered passive parameters and active conductance densities scales with the diameter to compensate for the altered axonal dimensions. For example, the decreased axonal diameter was compensated with increased Cm and decreased Ri while the larger axonal diameter was compensated by the markedly increased Ri. Nevertheless, the obtained Rm, Cm and Ri values remained in a biologically plausible range.

We would like to emphasize that these independent fitting steps ensured that the local biophysical environment will be similar irrespective of the morphology used, which resulted in comparable AP predictions.

We changed the title of this table to clarify the purpose of this data:

Table 2. Effect of axonal diameter and the Rseal on the model predictions
