# Supplementary figures and images for: Small Size of Recorded Neuronal Structures Confines the Accuracy in Direct Axonal Voltage Measurements
Source: eNeuro. 2021 Aug 2;8(4):ENEURO.0059-21.2021. doi: 10.1523/ENEURO.0059-21.2021 (PMC8342265; doi:10.1523/ENEURO.0059-21.2021)

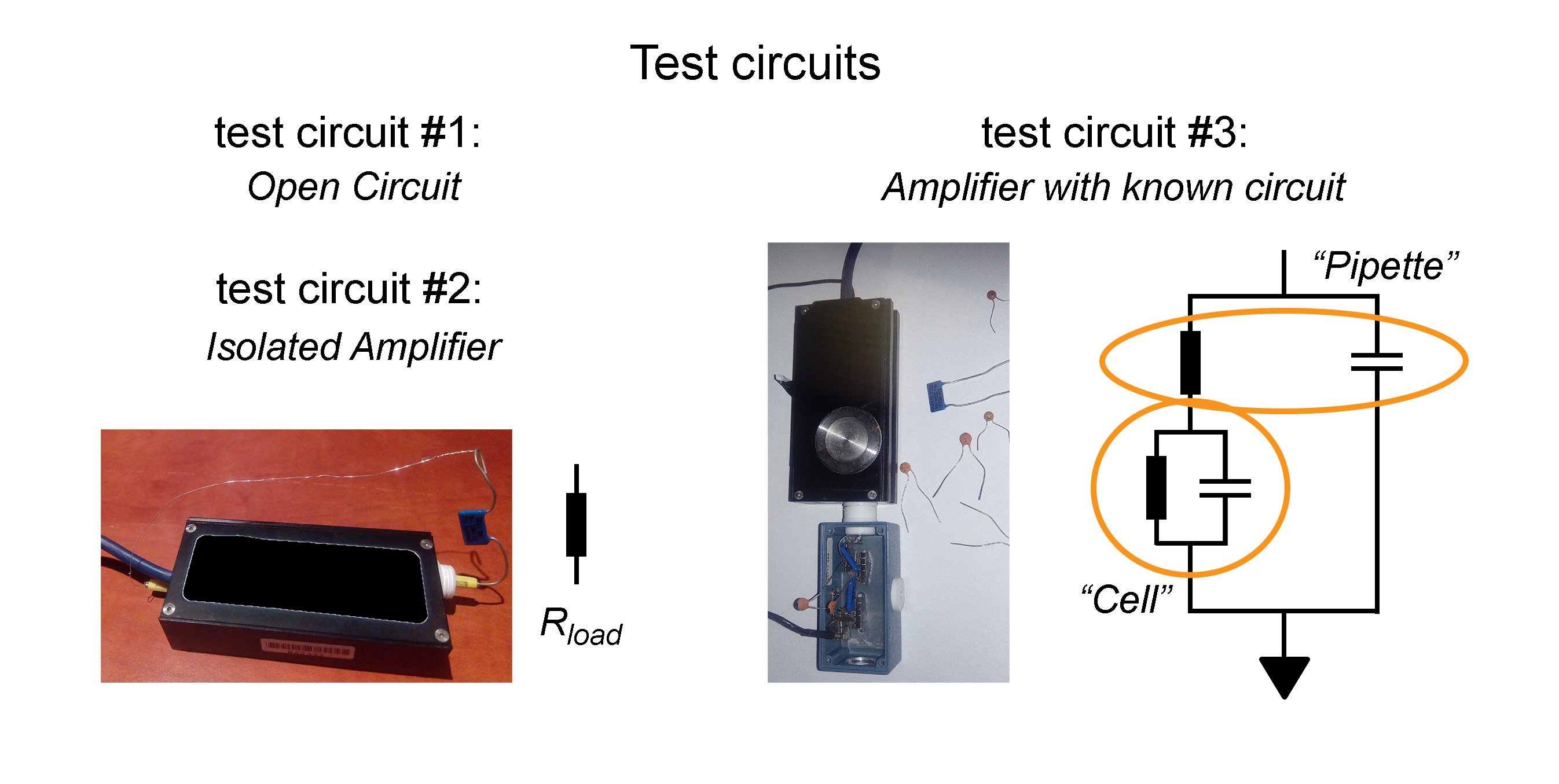

Supplement: Extended Data Figure 1-1 — Test circuits used for the characterization of the circuit components. Download Figure 1-1, TIF file. [file enu-eN-NWR-0059-21-s03.tif]

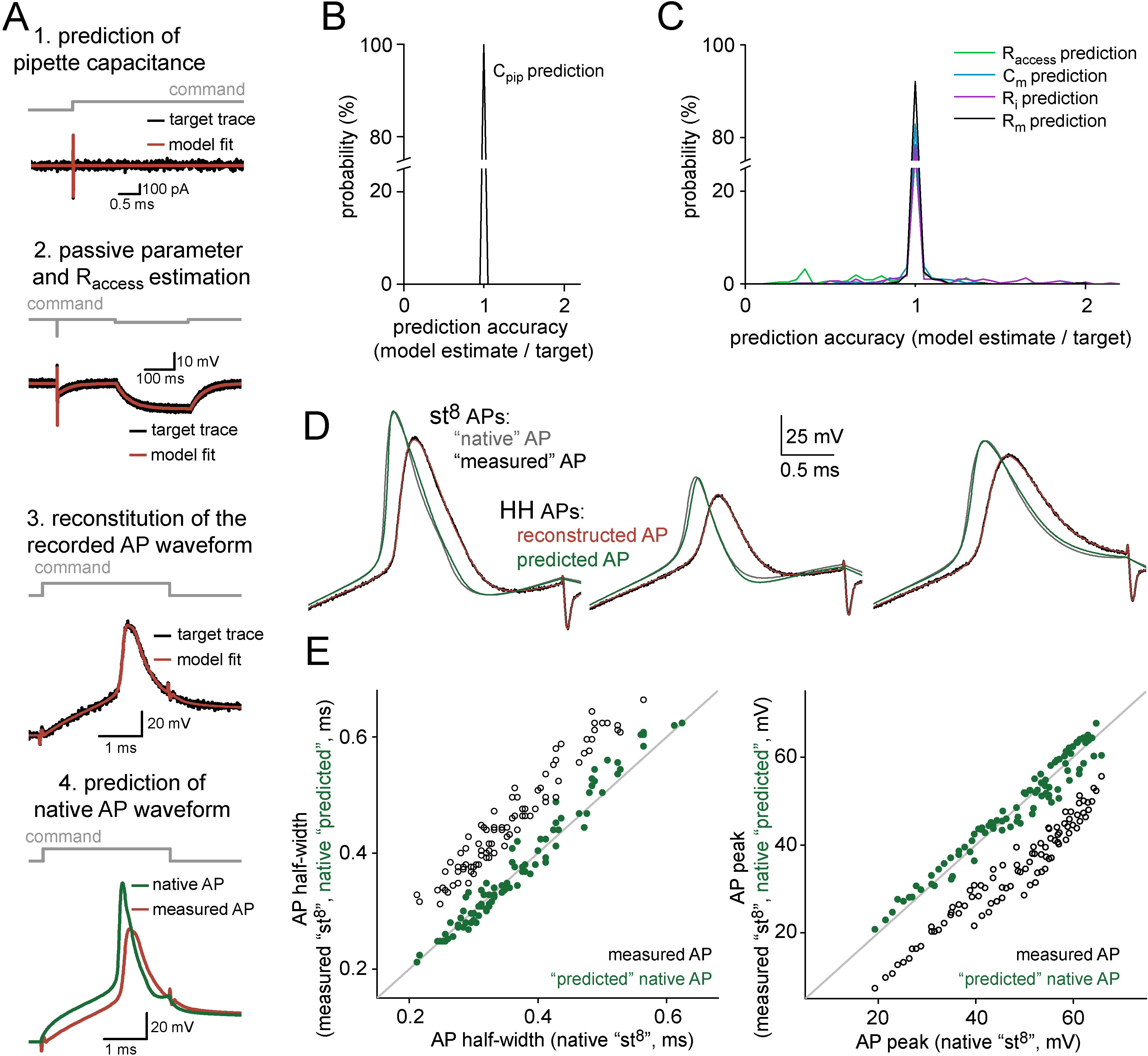

Supplement: Extended Data Figure 4-1 — Parameter extraction from recorded VC and CC data. A, Outline of the consecutive optimization steps that were used to retrieve the instrumental and the cellular parameters. B, Distribution of the relative error in Cpip estimations. C, Distribution of the relative errors present in the Raccess and passive parameter estimations. D, Overlay of representative APs (native and measured, gray and black traces, respectively) generated using 8-state active conductance models and corresponding best-fit APs generated with the standard conductances used in our final model (reconstructed and predicted, red and green traces, respectively). E, Comparison of measured AP half-width and peak values (black) and the corresponding predicted native parameters (green) with the original value (n = 90 simulated experiments). The line indicates equality. Download Figure 4-1, TIF file. [file enu-eN-NWR-0059-21-s04.tif]

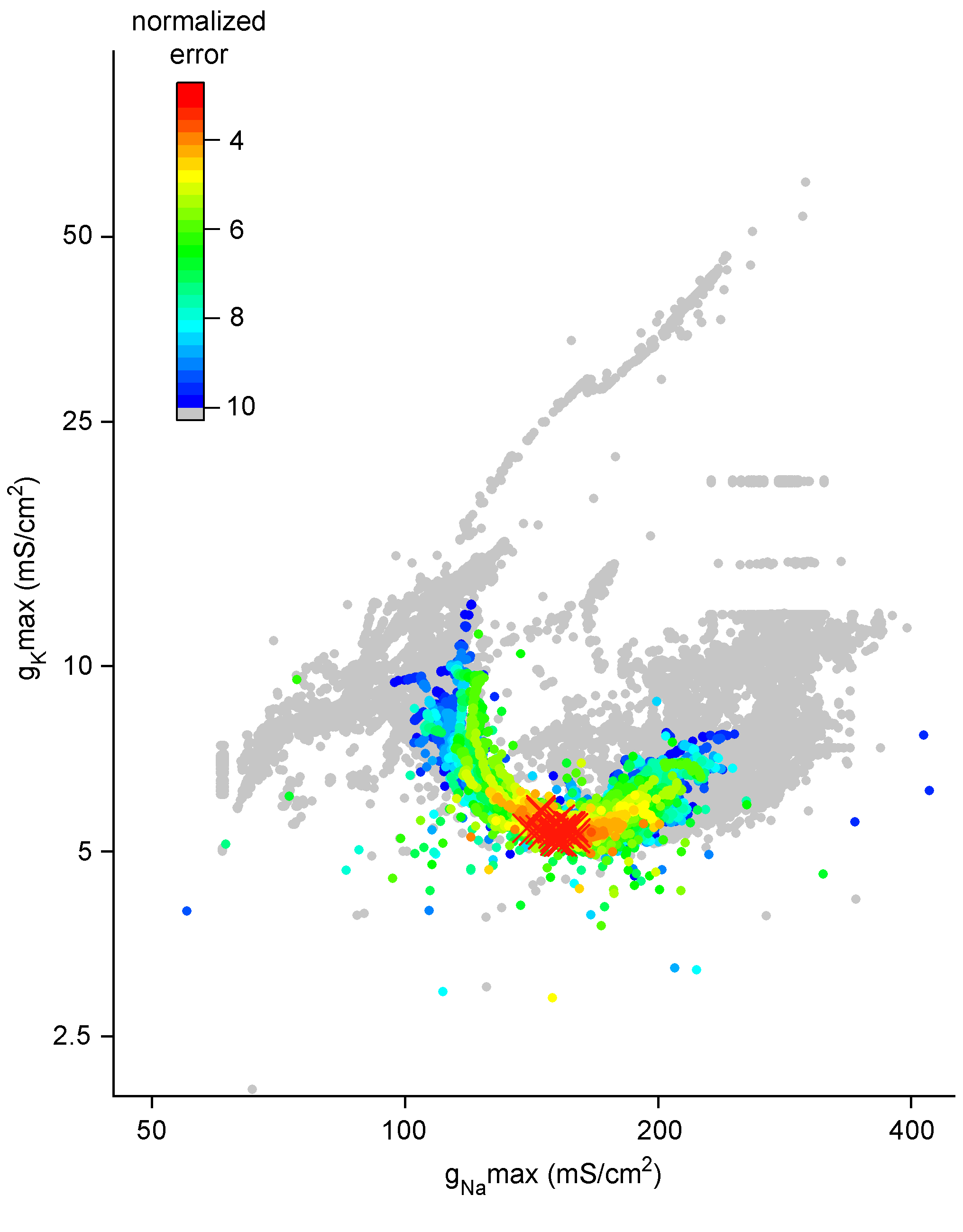

Supplement: Extended Data Figure 4-2 — Uniqueness of the fit results. The explored parameter space during the optimization of the conductances needed to reconstruct the recorded APs (30 fitted APs started from 120 initializations). Each dot represent single run during the fit (n = 142,605 runs). The optimization error of the each run is color coded. Red crosses mark best-fit solutions. Download Figure 4-2, TIF file. [file enu-eN-NWR-0059-21-s05.tif]

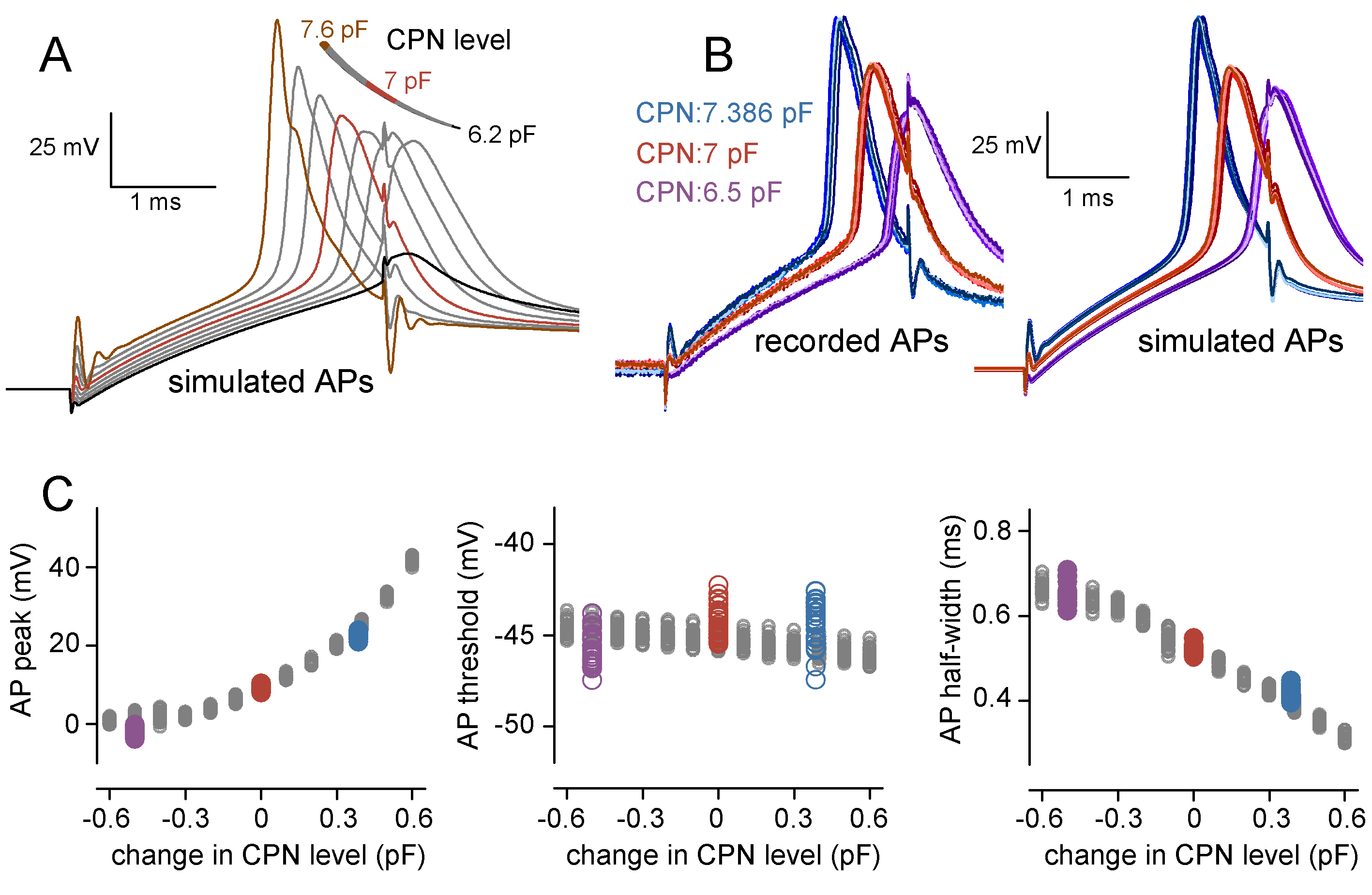

Supplement: Extended Data Figure 5-1 — Critical influence of the applied CPN on the recorded AP waveforms. A, Model AP waveforms simulated using the same conductance set but different CPN settings. Notice the AP failure at CPN = 6.2 pF (black) and the oscillation at CPN = 7.6 pF (brown). B, Representative APs recorded (left) and simulated (right) in the same axon with different CPN settings (n = 6 APs in each CPN conditions). C, Effects of different CPN level on AP peak (left), threshold (middle) and half-width (right). Gray circles show the AP parameters simulated with the 30 different conductance sets (the same set as in Figure 4). Purple, red and blue symbols show the experimentally recorded AP parameters obtained with three different CPN settings (n = 30 APs in each condition). Zero on x-axis represents the originally set 7 pF CPN. Download Figure 5-1, TIF file. [file enu-eN-NWR-0059-21-s06.tif]

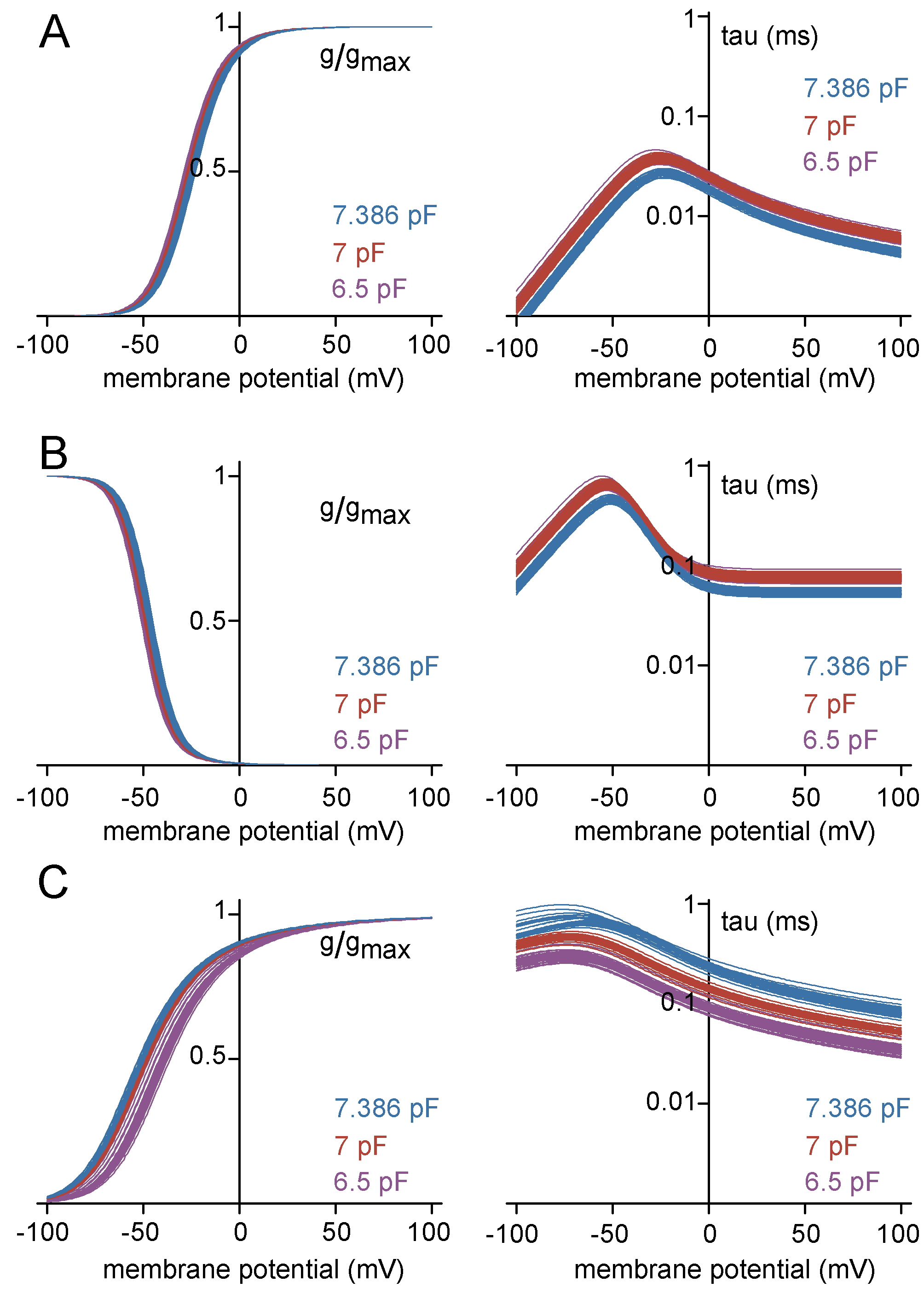

Supplement: Extended Data Figure 5-2 — Gating profile of model conductances obtained from fitting of AP waveforms with different levels of distortions. A, Voltage dependence (left) and kinetic profile (right) of the activation in Na+ conductance models obtained by the reconstruction of the APs recorded with different CPN settings (CPN = 6.5 pF in purple, CPN = 7 pF in red and CPN = 7.386 pF in blue). Each line represents single model conductance (n = 30/30/28 models). B, Same as in panel A but for the inactivation of the Na+ conductance models. C, Same as in panel A but for the K+ conductance models. Download Figure 5-2, TIF file. [file enu-eN-NWR-0059-21-s07.tif]

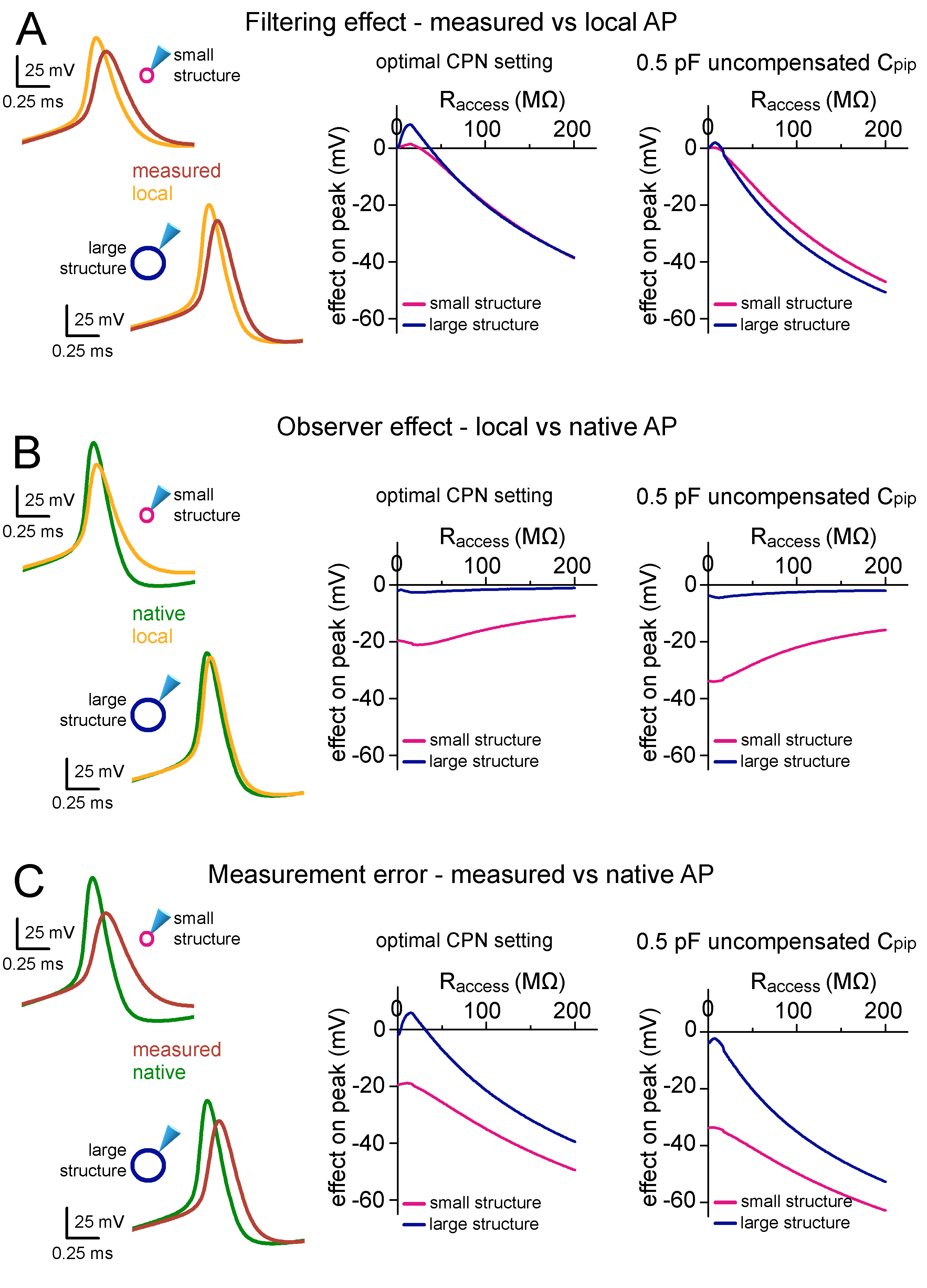

Supplement: Extended Data Figure 7-1 — Instrumental and structural parameters cooperatively determine signal distortions in recordings from small neuronal structures. The results of the same simulations as in Figure 7, are shown for effects on AP peak. Download Figure 7-1, TIF file. [file enu-eN-NWR-0059-21-s08.tif]
