# Supplementary material for: Small Size of Recorded Neuronal Structures Confines the Accuracy in Direct Axonal Voltage Measurements
Source: eNeuro. 2021 Aug 2;8(4):ENEURO.0059-21.2021. doi: 10.1523/ENEURO.0059-21.2021 (PMC8342265; doi:10.1523/ENEURO.0059-21.2021)
Supplement: Extended Data 1 — NEURON codes producing data shown in Figures 2, 5, 8. Download Extended Data 1, ZIP file. [file enu-eN-NWR-0059-21-s01.zip › realistic amplifier model/description of CC_circuit.pdf]

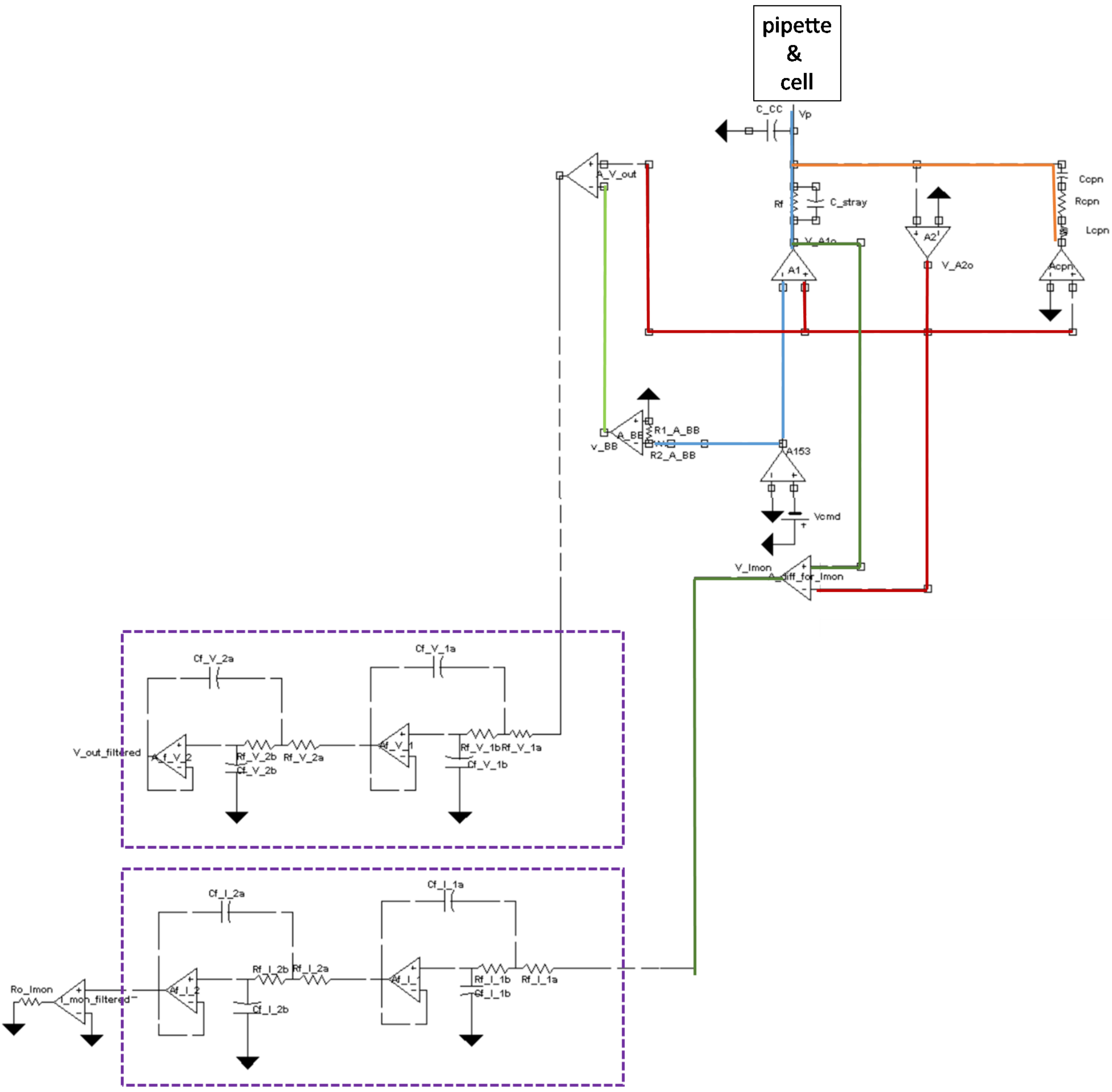

unity gain voltage follower (A2):

the op-amp that buffers the pipette potential.
Output signal (━) of A2 is fed to the capacitance neutralizing circuit, to the current generator circuit, to the current monitor circuit and to the differential amplifier, that generates bridge balance corrected output signal.

current generator circuit: ━━━━━━

As for VC, command potentials can be played by the **Vcmd** battery. To adjust the command, go to the LinearCircuit[0] → source(t)→Vcmd

alternatively, command can be edited within the CC.ses (line 26-36) this option allows the generation of additional command steps

The command signal is buffered by the **A153** op-amp, and injected to the pipette by the **A1** and **Rf** (Rf has stray capacitance, **Cstray**)

Note that because of the conversion factor of 0.5 V/nA (with 500 MOhm Rf), 1 mV command potential generates 0.5 pA current. To separate Rf from the pipette resistance output of A2 returns to the current generator path. (see Axon Guide, Chapter 3, A high-quality current source). A153 also provide the command signal for BB compensation

capacitance neutralization: ━━━━━━

A positive feedback loop that injects the output signal of the voltage follower to the Vp, by the **Ccpn** capacitor. Level of capacitance neutralization can be set by adjusting the gain of **Acpn. Lcpn** and **Rcpn** are parasitic elements that makes the signal artefacts associated with the capacitance neutralization more realistic.
Capacitance neutralization can be adjusted in the model by:

set_CPN($01) //the argument is the desired compensation in pF

bridge balance compensation: ━━━━━━

A_BB generates a scaled version of the command signal.
Bridge balance can be adjusted in the model by:

set_BB ($01) //the argument is the desired compensation in Mohm

A_V_out is a differential op-amp that generates the final, bridge balance corrected output voltage. Output of this path goes to a 4-pole bessel filter. The output of the filtering cascade is the monitored voltage output of the model (**V_out_filtered**).

current monitor: ━━━━━━

A_diff_for_Imon re-creates the actual voltage command. Output of this path goes to a 4-pole bessel filter. Following the filtering cascade, the current passing through the **I_mon_filtered** op-amp is the monitored output current of the model (**I_mon_filtered**).

Bessel filter: - - - - - - - - - -

Current and voltage outputs have separate filter chains. Parameters of the filters were set to create 4-pole Bessel characteristics. Cutoff frequency of the filters can be adjusted with:

set_filter($01, $02) //1st argument is the desired cutoff frequency for the V_output in kHz , the second one is the same for the I_output.

(0.5, 1, 2, 3, 4, 6, 8, 10, 12, 14, 16, 18, 20, 22, 24, 26, 28, 30, 100 kHz filters are implemented).

Ccc sets the intrinsic capacitance of the CC circuit to its observed level.
