# Supplementary material for: Small Size of Recorded Neuronal Structures Confines the Accuracy in Direct Axonal Voltage Measurements
Source: eNeuro. 2021 Aug 2;8(4):ENEURO.0059-21.2021. doi: 10.1523/ENEURO.0059-21.2021 (PMC8342265; doi:10.1523/ENEURO.0059-21.2021)
Supplement: Extended Data 1 — NEURON codes producing data shown in Figures 2, 5, 8. Download Extended Data 1, ZIP file. [file enu-eN-NWR-0059-21-s01.zip › realistic amplifier model/description of VC_circuit.pdf]

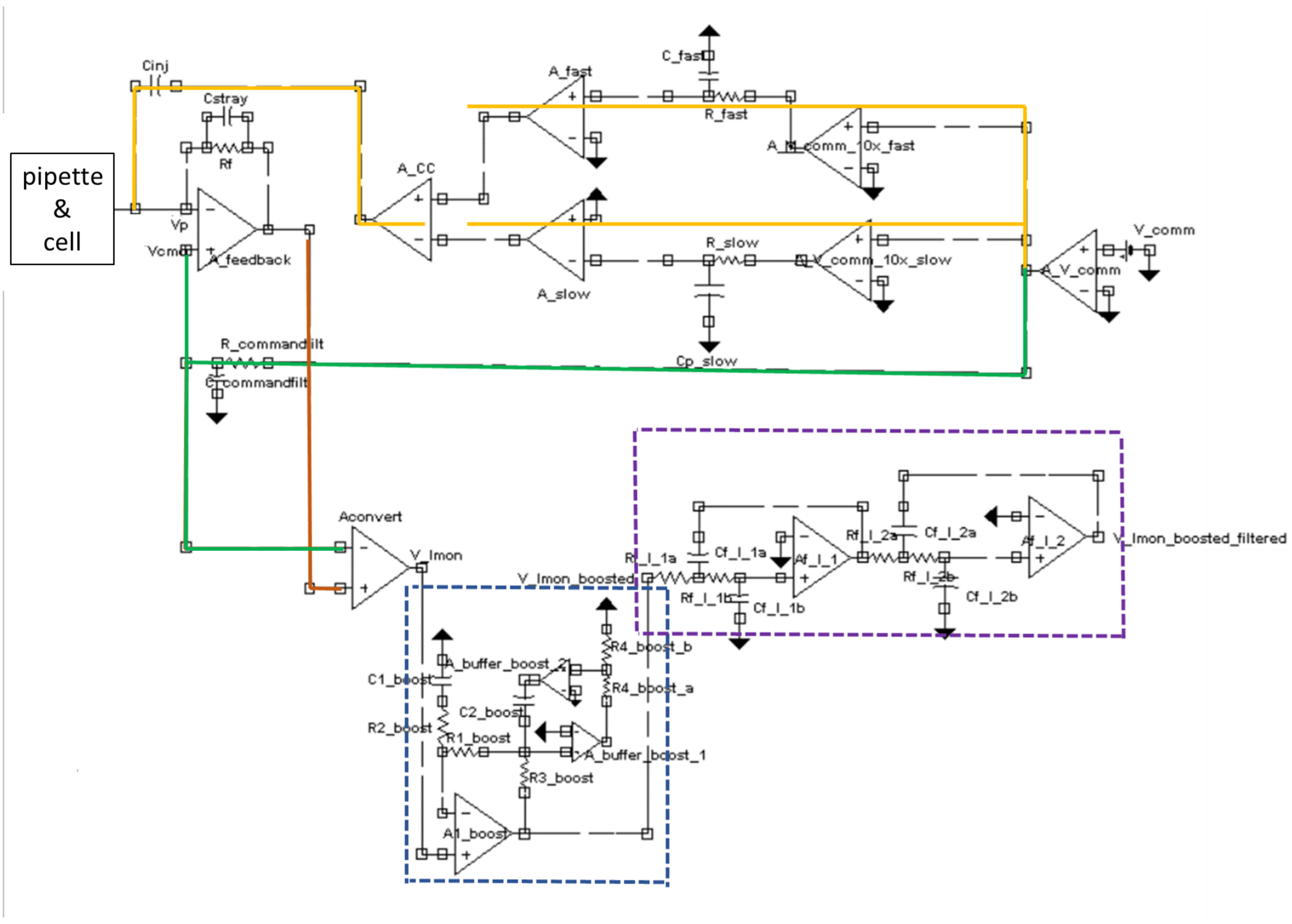

command generator:

Command potentials can be played by the **V_comm** battery (on the left) . To adjust the command, go to the LinearCircuit[0] → source(t)→V_comm

alternatively, command can be edited within the VC.ses (line 27-30) this option allows the generation of additional command steps

The command signal is injected by the **A_V_comm** noninverting op-amp. This op-amp has a time constant of 10 us.

Speed of the command signal should approximately match with the time constant formed by the RC circuit of the pipette. We introduced **R_commandfilt** and **C_commandfilt** which form a lowpass filter with 0.95 us tau.

capacitance compensation :

In general, scaled version of the V_comm is injected by the **Cinj** to correct the pipette potential (**Vp**). Magnitude and temporal profile of the compensation should adjust until pipette related capacitive transients disappear. We implemented two separate circuits, as Multiclamp offers both fast and slow pipette capacitance compensation.
Cpfast:
A_V_comm_10x_fast buffers the command. **R_fast** and **C_fast** determine the temporal characteristics of the compensatory signal. **A_fast** set the magnitude of fast compensation.
Cpslow:
A_V_comm_10x_slow buffers the command. **R_slow** and **C_slow** determine the temporal characteristics of the compensatory signal. **A_slow** set the magnitude of fast compensation.
A_CC feeds the combination of the two compensatory current by the **Cinj** to the **Vp**

Capacitance compensation can be adjusted by:
set_Cp_fast($01,$02) //1st argument is the desired compensation in pF, 2nd arg is the time constant for compensation
set_Cp_slow($01,$02) //1st argument is the desired compensation in pF, 2nd arg is the time constant for compensation
set_Cp_comp($01,$02, $03,$04) //combined Cpfast/Cpslow compensation. It has four arguments: fast magnitude, fast tau, slow magnitude, slow tau

I-V converter:

Signal from the resistive feedback circuit (**A_feedback** with **Rf** and **Cstray**) and the command signal are fed to the differential amplifier (**A_convert**) that generates signal proportional to the clamp current. 500 MOhm Rf needs a conversion factor of 0.5 V/nA. If A_convert gain is 2, output of the differential amplifier (**V_Imon**) will be in pA.

High frequency boost:

The RC circuit formed by Rf and Cstray slows down the response speed of V_Imon. We adjusted the experimentally observed response speed using the high frequency boost circuit. Output of **A1_boost** is the boosted current signal, **V_Imon_boosted**. Note that the behavior of the I-V converter (and thus the V_Imon responses) will be ideal after the removal of Cstray (Cstray=0).

Bessel filter:

V_Imon_boosted is the input of the two cascaded Sallen-key filter chain. Parameters of the filter were adjusted to create 4-pole bessel characteristics. Cutoff frequency of the filter can be adjusted with:
set_filter() //argument is the desired cutoff frequency in kHz.
(0.5, 1, 2, 3, 4, 6, 8, 10, 12, 14, 16, 18, 20, 22, 24, 26, 28, 30, 100 kHz filters are implemented).
V_Imon_boosted_filered is the output of the filter.

FURTHER READING:
Sigworth, 1995, Electronic Design of the Patch Clamp, in: Single-Channel Recording, Chapter 4
